# Molecular Mapping of Reduced Plant Height Gene *Rht24* in Bread Wheat

**DOI:** 10.3389/fpls.2017.01379

**Published:** 2017-08-08

**Authors:** Xiuling Tian, Weie Wen, Li Xie, Luping Fu, Dengan Xu, Chao Fu, Desen Wang, Xinmin Chen, Xianchun Xia, Quanjia Chen, Zhonghu He, Shuanghe Cao

**Affiliations:** ^1^College of Agronomy, Xinjiang Agricultural University Urumqi, China; ^2^Institute of Crop Science, National Wheat Improvement Center, Chinese Academy of Agricultural Sciences Beijing, China; ^3^International Maize and Wheat Improvement Center (CIMMYT) Beijing, China

**Keywords:** CAPS marker, genome mining, *Rht24*, SNP chip, *Triticum aestivum*

## Abstract

Height is an important trait related to plant architecture and yield potential in bread wheat (*Triticum aestivum* L.). We previously identified a major quantitative trait locus *QPH.caas-6A* flanked by simple sequence repeat markers *Xbarc103* and *Xwmc256* that reduced height by 8.0–10.4%. Here *QPH.caas-6A*, designated as *Rht24*, was confirmed using recombinant inbred lines (RILs) derived from a Jingdong 8/Aikang 58 cross. The target sequences of *Xbarc103* and *Xwmc256* were used as queries to BLAST against International Wheat Genome Sequence Consortium database and hit a super scaffold of approximately 208 Mb. Based on gene annotation of the scaffold, three gene-specific markers were developed to genotype the RILs, and *Rht24* was narrowed to a 1.85 cM interval between *TaAP2* and *TaFAR*. In addition, three single nucleotide polymorphism (SNP) markers linked to *Rht24* were identified from SNP chip-based screening in combination with bulked segregant analysis. The allelic efficacy of *Rht24* was validated in 242 elite wheat varieties using *TaAP2* and *TaFAR* markers. These showed a significant association between genotypes and plant height. *Rht24* reduced plant height by an average of 6.0–7.9 cm across environments and were significantly associated with an increased TGW of 2.0–3.4 g. The findings indicate that *Rht24* is a common dwarfing gene in wheat breeding, and *TaAP2* and *TaFAR* can be used for marker-assisted selection.

## Introduction

Bread wheat (*Triticum aestivum* L.) is one of the most important food crops providing a significant proportion of the calories and protein consumed by humans (Mir et al., 2012; Chen et al., 2016). Grain yield in wheat largely depends on plant architecture, particularly plant height, which is significantly associated with biomass production and harvest index that ultimately determine yield potential (Law et al., 1978). Most importantly, appropriately reduced plant height reduces lodging and increases grain yield (Evans, 1998). The introduction of semi-dwarf varieties leading to the “Green Revolution” greatly enhanced crop yields globally (Peng et al., 1999).

To date, 23 dwarfing genes (*Rht1*–*Rht23*) have been cataloged in wheat (Supplementary Table S1) (McIntosh et al., 2015). Among them, only four homeologous genes *Rht1* (*Rht-B1b*), *Rht2* (*Rht-D1b*), *Rht3* (*Rht-B1c*), and *Rht10* (*Rht-D1c*) from the chromosome 4BS, 4DS, 4BS, and 4DS, respectively, had been cloned (Peng et al., 1999; Pearce et al., 2011; Wu et al., 2011), 13 were on chromosomes 2AS (2), 2BL (1), 2DL (1), 3BS (1), 5AL (1), 5DL (1), 6AS (3), 7AS (1), and 7BS (2), but the locations of six other dwarfing genes have not been determined yet (Peng et al., 1999, 2011; Ellis et al., 2005; Wu et al., 2011; McIntosh et al., 2013; Chen et al., 2015). So far, more than 50 quantitative trait loci (QTL) for plant height have also been identified on all wheat chromosomes in previous reports (Börner et al., 2002; Peng et al., 2003; Quarrie et al., 2005; Liu et al., 2011; Griffiths et al., 2012; Würschum et al., 2015). For example, Wei et al. (2010) identified quite a few stable plant height QTL located on chromosomes 2A, 2B, 2D, 3B, 4B, 5A, 5D, 7B, and 7D. Griffiths et al. (2012) found that all chromosomes except for 3D, 4A, and 5D, had plant height genes using meta-QTL analysis. However, only a few genes for reduced stature have been used in wheat breeding because most showed negative effects on grain yield (Law et al., 1978; Worland et al., 1998; Chen et al., 2015). *Rht-B1b* (4BS), *Rht-D1b* (4DS) and *Rht8* (2DL) are extensively used in wheat breeding globally and molecular markers have been developed for their marker-assisted selection (MAS) (Korzun et al., 1998; Peng et al., 1999; Rebetzke and Richards, 2000; Ellis et al., 2002; Asplund et al., 2012). *Rht-B1b* (*Rht1*) and *Rht-D1b* (*Rht2*) were two major genes in the Green Revolution, and at present, approximately 70% of wheat varieties throughout the world contain at least one of them (Evans, 1998). *Rht8* reduced plant height around 10% and it has not significant negative effect on grain yield (Worland et al., 1998).

Simple sequence repeat (SSR) markers were favored for research and breeding in the last two decades due to high polymorphism, good repeatability, co-dominance and a simple polymerase chain reaction (PCR)-based system for analysis (Röder et al., 1998). With rapid advancements in sequencing technology, the quality of wheat genome assembly has been significantly improved and large numbers of high-quality scaffolds to facilitate gene isolation are available (Rogers, 2014). Simultaneously, many single nucleotide polymorphisms (SNPs) were identified for developing SNP chips, providing high-throughput genotyping platforms that have been extensively used for genome-wide association studies (GWAS) and QTL mapping at high resolution (Colasuonno et al., 2014; Wang et al., 2014; Sukumaran et al., 2015; Gao et al., 2016; Wu et al., 2016). Many QTL for agronomic traits, grain and industrial quality and disease resistance have been identified in wheat (Buerstmayr et al., 2009; Wang et al., 2014; Naruoka et al., 2015; Jin et al., 2016; Liu et al., 2016; Zhai et al., 2016). Thus, the availability of high quality wheat genome data and high-throughput SNP genotyping platforms greatly advanced wheat genetics and breeding.

We previously identified a plant height major QTL *QPH.caas-6A* between *Xwmc256* and *Xbarc103*, which explained 8.0–10.4% of the phenotypic variance across eight environments using an *F*_2:4_ population derived from the Jingdong 8/Aikang 58 cross (Li et al., 2015). In addition, *QPH.caas-6A* probably was consistent with *QTL_height_6A_1*, which also linked with *Xwmc256* on chromosome 6AL and explained 6.3–29.1% of the phenotypic variance (PVE) (Griffiths et al., 2012). Furthermore, *QPH.caas-6A* was identified to reduce TGW 6.5–8.2%, increase kernel number per spike and number of spike by 2.4–3.5% and NS by 2.0–4.6%, respectively, which is similar to “green revolution” gene *Rht2* (Li et al., 2015). Thus *QPH.caas-6A* was a potentially useful dwarfing locus in wheat breeding. Here we designate *QPH.caas-6A* as *Rht24*. The previously closest markers flanking *Rht24* were *Xbarc103* and *Xwmc256*, which were 8.2 cM apart. The aim of the present study was to identify markers more closely linked to *Rht24* using the wheat genomic database and a 660K SNP chip and thereby establish a more efficient MAS system for wheat breeding.

## Materials and Methods

### Plant Materials

Two hundred and fifty-six recombinant inbred lines (RILs, *F*_2:6)_ from a cross between Aikang 58 (AK58) × Jingdong 8 (JD8) were used for genetic analysis. AK58, a leading variety occupying more than one million ha in the Yellow and Huai Valley, has a short plant height and excellent lodging resistance. Jingdong 8, with relatively tall plant height and excellent resistance to heat during the grain filling stage, was an elite variety in the Northern China Plain Region. Two sets of Chinese and introduced elite varieties (Supplementary Tables S4, S5), comprising 154 (Set I) and 88 (Set II) varieties, were used to validate the efficacy of MAS for *Rht24* and to investigate allelic distributions.

### Field Trials and Phenotype Evaluation

The RILs and parents were planted at Gaoyi in Hebei province and Anyang in Henan during the 2014–2015 cropping season, and at Gaoyi during 2015–2016 cropping season. The experimental design was randomized complete blocks and three replications. Each plot was a single 2 m row with 25 cm between rows.

Set I varieties were planted at Anyang in Henan province and Suixi in Anhui, respectively, during the 2012–2013 and 2013–2014 cropping seasons. Set II varieties were grown at Shijiazhuang in Hebei and Beijing, respectively, during the 2012–2013 and 2013–2014 cropping seasons. These were grown in randomized complete blocks with three replications. Each plot consisted of four 2 m rows spaced 30 cm apart and approximately 50 plants in each row. Field management was according to local practice. During the whole wheat growth period in our field trial, there is no extreme weather causing serious damages, such as cold spell in later spring, dry and hot wind in filling stage, and fertilization (bottom fertilizer including 150 kg/h^2^ Urea plus 400 kg/h^2^ Diammonium Phosphate; 300 kg/h^2^ Urea just before elongation stage) is enough for wheat growth and development.

Plant height was measured from the ground to spike (awns excluded) at grain-filling. For each plot, five representative primary tillers on different plants in the middle of each row were selected to measure the plant height, and the averaged value was used for subsequent analysis. TGW was determined by weighing triplicate 200 grain samples.

### Genotypic Analysis Using Simple Sequence Repeat (SSR) Markers

Genomic DNA was isolated from young leaves using the modified CTAB method (Saghai-Maroof et al., 1984). Twenty-two SSR markers on chromosome 6AL were chosen for genetic analyses^1^ (Supplementary Table S7).

Polymerase chain reaction was performed in a 15 μl reaction system containing 7.5 μl of 2 × Taq PCR Mix (Tianwei Biotechnology Co., Ltd., Beijing^2^), 5 pmol of each primer and 100 ng of genomic DNA. Amplification was performed at 94°C for 5 min, followed by 35 cycles at 94°C for 20 s, 50–60°C (depending on specific primers) for 30 s and 72°C for 1 min, with a final extension at 72°C for 5 min. The PCR products were separated in 6% polyacrylamide or 2% agarose gels.

### Gene-Specific Marker Development by Genome Mining Approach

To confirm the target sequences of the SSR mentioned above, PCR products were purified using the TIANgel MIDI Purification Kit (Tiangen, Biotechnology Co., Ltd., Beijing^3^). The purified PCR products were ligated with cloning vector pEASY-T5 Zero and then transformed into Trans1-T1 competent cells by the heat shock method (TransGen Biotech Co., Ltd., Beijing^4^). At least three positive clones from each transformation were randomly selected and sequenced at Shanghai Sangon Biotech Co., Ltd^5^. The SSR target sequences were used as BLAST queries^6^ to identify desirable scaffolds. The genes of interest in the target scaffold were isolated and sequenced following the above procedure. Sequence sites polymorphic between the parents were identified by alignments with DNAMAN software^7^ for designing gene-specific markers. Cleaved amplified polymorphic sequences (CAPS) or derived CAPS (dCAPS) marker for each target gene was developed following Thiel et al. (2004).

### SNP Chip-Based Screening

Wheat 660K SNP chip was developed by the Institute of Crop Science, Chinese Academy of Agricultural Sciences, synthesized by Affymetrix and commercially available at CapitalBio Corporation^8^. Recently, the chip has been efficiently used in our lab (Jin et al., 2016).

To identify more molecular markers linked to *Rht24* the 660K SNP chip was used to test two parents and two contrasting bulks of the RIL population, comprising equal amounts of genomic DNA from 10 tall and 10 short RILs, respectively. Genotyping was performed at CapitalBio Technology Company^9^. SNPs polymorphic between parents and between two bulks were selected to develop CAPS markers.

### Genetic Linkage Map Construction and Statistical Analysis

Quantitative trait locus IciMapping 4.0^10^ was used for linkage map construction and QTL analysis using a LOD score threshold of 3.0 (Lincoln et al., 1992). Plant height was reformed with an algorithm SERiation and criterion SARF (sum of adjacent recombination frequencies). Variance analysis and *t*-test (Duncan method) were conducted with SAS 9.4^11^. The broad-sense heritability (*h*_B_^2^) of the corresponding traits was calculated using the following formula:

$${h_{B}}^{2}=\sigma^{2}g/({\sigma^{2}}_{g}+{\sigma^{2}}_{ge}/e+{\sigma^{2}}_{\varepsilon }/re),$$

where σ^2^g, σ^2^_ge_, and σ^2^_ε_ were estimates of lines, line × environment interactions and residual error variances, respectively, and *e* and *r* represented the numbers of environments and replicates, respectively (Supplementary Table S6; Nyquist and Baker, 1991). *T*-test was used to perform the association analysis between *Rht24* and plant height as well as TGW.

## Results

### Confirmation of *Rht24*

In our previous study, *Rht24* was identified with LOD score of approximately 12, explaining 8.0–10.4% of the phenotypic variance; it was mapped within an 8.2 cM interval flanked by *Xbarc103* and *Xwmc25*6 (Li et al., 2015). To further verify *Rht24*, 22 SSR markers were selected from the targeted region of chromosome 6AL (Supplementary Table S7). Among them, only *Xbarc103* and *Xwmc256* were polymorphic between the parental lines and bulk lines built by 10 tall and 10 short lines (Supplementary Figure S1). *Rht24* was located between the two markers, which were 19.46 cM apart. Although the genetic distance was greater than reported previously, the QTL for reduced plant height was confirmed.

### Narrow-Down of *Rht24* by the Genome Mining Approach

**FIGURE 1:**
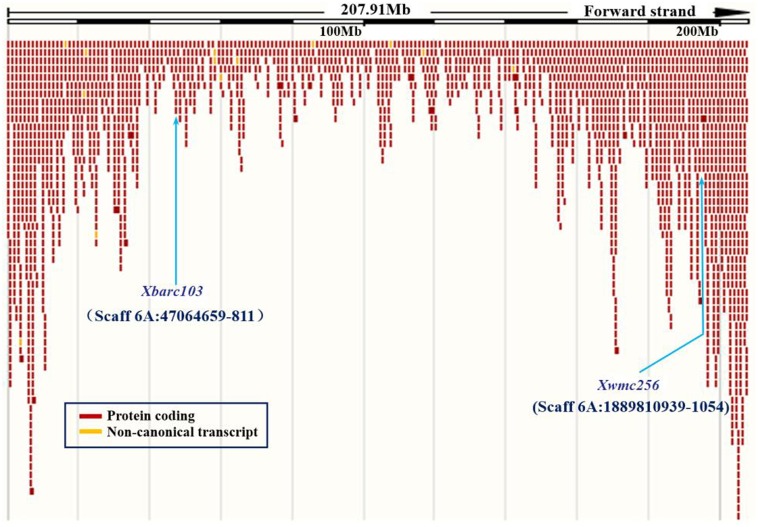
A super scaffold spanning *Rht24*. Locations of SSR loci *Xbarc103* and *Xwmc256* are shown by *arrows*.

**FIGURE 2:**
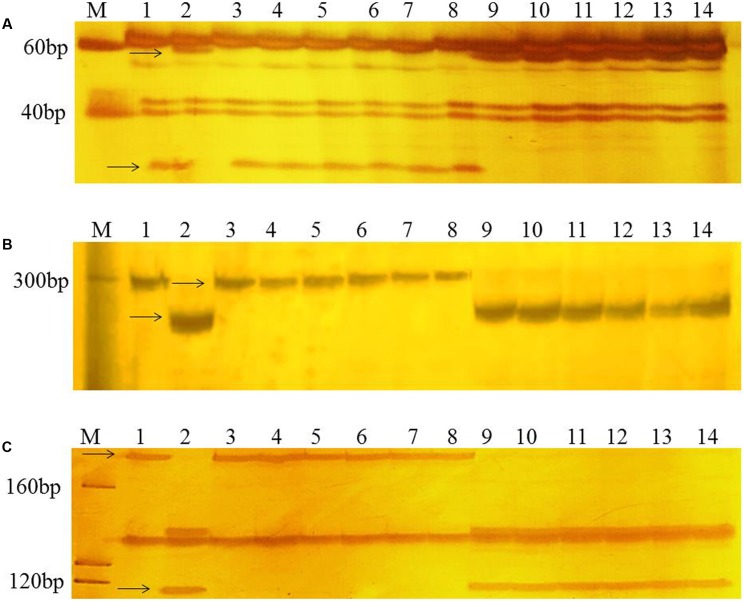
PCR patterns of polymorphic markers *TaGA3* **(A)**, *TaFAR* **(B)**, and *TaAP2* **(C)**. Polymorphic fragments associated with height differences between short and tall RILs are shown by *arrows*. M, Marker (20 bp DNA ladder, Takara Bio Company). *Lanes 1* and *2* are the parents, AK58 and JD8, respectively; *lanes 3–8* are short RILs; *lanes 9–14* are tall RILs.

**FIGURE 3:**
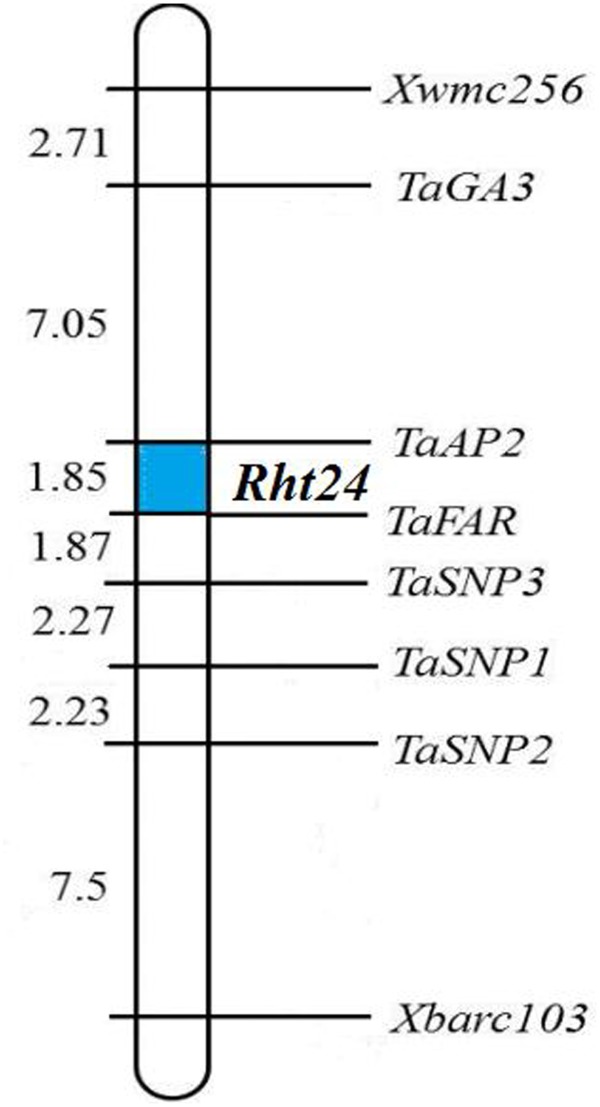
Linkage map of *Rht24*. The names and corresponding locations of all markers are indicated on the *right* side of the map. Genetic distance (cM) is shown on the *left* side of the map. The region of *Rht24* is highlighted in *blue*.

To confirm the chromosomal location of *Rht24*, we cloned and sequenced the differential fragments of *Xbarc103* and *Xwmc256* in the tall and short lines, respectively (Supplementary Figure S1). Sequencing results showed that they were consistent with the reported sequences^12^. Using the sequences as queries to BLAST against International Wheat Genome Sequence Consortium database^13^, a super scaffold of approximately 208 Mb (approximately 1200 genes) spanning *Rht24* was identified (**Figure 1**). Twenty genes involved in plant morphogenesis and hormone metabolism were selected from the scaffold and sequenced (Supplementary Table S8). Polymorphisms between AK58 and JD8 were detected at loci *TaGA3*, *TaFAR*, and *TaAP2* (Supplementary Figures S2A–C and Table S2). Based on these polymorphisms, three gene-specific CAPS or dCAPS markers were developed (**Figures 2A–C** and Supplementary Figures S2A–C). Upon genotyping the RIL, the location of *Rht24* was narrowed to a 1.85 cM interval flanked by *TaFAR* and *TaAP2* (**Figure 3**).

### Enriching Molecular Markers in the *Rht24* Region by a Combination of SNP Genotyping Assays and Genomic Identification

To enrich molecular markers in the *Rht24* region, the 660K SNP chip was used in genotyping assays. In total, more than 400 polymorphic SNPs on chromosome 6A from wheat 660K SNP chips were identified between two contrasting bulks (data not shown). Among them, the target sequences of polymorphic SNPs anchored in the super scaffold above were used to develop site-specific CAPS markers (Supplementary Table S9). Among them, three polymorphic loci, *TaSNP1*, *TaSNP2*, and *TaSNP3*, were mapped in the region of *Rht24* (Supplementary Figures S3A–C, S4A–C, Table S2 and **Figure 3**). However, they did not further narrow down the *Rht24* locus.

### Validation of Molecular Markers Closely Linked to *Rht24*

Among 242 wheat varieties genotyped by markers *TaFAR* and *TaAP2* (Supplementary Figures S5A,B), *Rht24* was present in 185 (76.4%) varieties and absent in 41 (17%) (Supplementary Table S3). The average plant height for the varieties containing *Rht24* was 78.2 and 80.2 cm in Sets I and II, respectively, whereas for those without *Rht24* the comparative values were 86.1 and 86.2 cm, respectively, indicating that varieties with *Rht24* were generally shorter than those without (*P*_I_ = 0.029, *P*_II_ = 0.003, **Table 1**). *Rht24* was also significantly associated with thousand grain weight (TGW) in Set I (*P* = 0.014, **Table 2**). Although *Rht24* had no significant association with TGW in Set II, the average TGW of entries with *Rht24* was 2 g higher than those without (*P* = 0.196, **Table 2**). On average *Rht24* reduced plant height by 6.0–7.9 cm and increased TGW by 2.0–3.4 g in all varieties used in this study (**Tables 1**, **2**).

**Table 1:** Association analysis between *Rht24* genotypes and plant height in two sets of germplasm.

Germplasm	Year	Environment	Genotype^a^	Number of accessions	Mean plant height (cm)^b^	*SD* (cm)	Range (cm)
Set I	2012–2013	Anyang	*A*	17	88.5a	14.7	75.0–138.3
			*B*	128	81.0a	7.0	63.3–106.7
		Suixi	*A*	17	84.8a	12.4	71.0–125.0
			*B*	128	77.2b	6.6	55.0-95.7
	2013–2014	Anyang	*A*	17	83.6a	15.2	66.0–135.7
			*B*	128	74.4b	7.0	58.2–92.1
		Suixi	*A*	17	87.8a	12.4	72.4–125.8
			*B*	128	80.2b	6.6	59.8–100.9
		Average	*A*	17	86.1a	13.5	72.2–131.2
			*B*	128	78.2b	6.4	59.5–98.8

Set II	2012–2013	Beijing	*A*	24	82.3a	10.3	62.9–109.8
			*B*	57	76.0b	8.0	49.3–93.9
		Shijiazhuang	*A*	24	85.3a	9.0	68.2–108.4
			*B*	57	80.5b	7.6	55.6–97.3
	2013–2014	Beijing	*A*	24	82.3a	10.3	62.9–109.8
			*B*	57	76.0b	8.0	49.3–93.9
		Shijiazhuang	*A*	24	85.3a	9.0	68.2–108.4
			*B*	57	80.5b	7.6	55.6–97.3
		Average	*A*	24	86.2a	9.5	67.8–110.7
			*B*	57	80.2b	7.5	57.7–95.7


*^a^*A*, JD8 parental genotypes and *B*, AK58 parental genotypes based on flanking markers *TaAP2* and *TaFAR*. Varieties with recombinant genotypes are not included.*

*^b^Different lowercase letters following the mean plant height indicate significant differences between two genotypes at *P* < 0.05 (Dunnett’s *t*-test); SD, standard deviation.*

**Table 2:** Association analysis between *Rht24* genotypes and TGW in two sets of germplasm.

Germplasm	Year	Environment	Genotype^a^	Number of accessions	Mean TGW (g)^b^	*SD* (g)	Range (g)
Set I	2012–2013	Anyang	*A*	17	38.7a	6.0	30.1–53.3
			*B*	128	41.4b	5.0	25.3–56.1
		Suixi	*A*	17	39.2a	6.1	29.3–49.8
			*B*	128	42.7b	5.5	25.7–56.3
	2013–2014	Anyang	*A*	17	47.0a	6.9	35.3–59.7
			*B*	128	50.3b	5.2	28.3–63.4
		Suixi	*A*	17	42.4a	7.4	30.2–57.7
			*B*	128	46.3b	5.7	22.5–62.2
		Average	*A*	17	41.8a	6.3	31.7–54.0
			*B*	128	45.2b	5.0	26.1–59.2

Set II	2012–2013	Beijing	*A*	24	30.7a	6.6	21.5–43.3
			*B*	57	32.2a	5.5	21.4–42.9
		Shijiazhuang	*A*	24	32.3a	7.1	20.9–48.1
			*B*	57	34.1a	6.0	21.1–46.3
	2013–2014	Beijing	*A*	24	29.6a	6.0	20.5–44.4
			*B*	57	30.9a	4.9	19.7–40.1
		Shijiazhuang	*A*	24	39.1a	9.4	25.5–66.1
			*B*	57	42.3a	7.7	26.3–56.3
		Average	*A*	24	32.9a	7.1	22.1–50.5
			*B*	57	34.9a	5.7	22.5–46.1


*^a^A, *FAR-a*/*AP2-a* (parental JD8 genotype); B, *FAR-b*/*AP2-b* (parental AK58 genotype). ^b^Different lowercase letters following the mean plant height indicate significant differences between two genotypes at *P* < 0.05 (Dunnett’s *t*-test); SD, standard deviation.*

## Discussion

### *Rht24* Is an Important Locus Affecting Plant Height and Grain Weight in Wheat

Previous studies showed that loci controlling plant height were present in all 21 wheat chromosomes (Mccartney et al., 2005; Gale and Law, 1973; Griffiths et al., 2012). The QTL *QTL_height_6A_1* linked with *Xwmc256* on chromosome 6AL explained 6.3–29.1% of the phenotypic variance (PVE) (Griffiths et al., 2012). *Rht24*, previously designated as *QPH.caas-6A*, was flanked by SSR markers *Xwmc256* and *Xbarc103* (Li et al., 2015), indicating that it could be the same as *QTL_height_6A_1*. Here we developed two markers, *TaFAR* and *TaAP2* that closely flanked *Rht24*. The two markers were used to test the genetic effect of *Rht24* and observed similar result with the previously reported (**Table 3**; Griffiths et al., 2012; Li et al., 2015). *Rht24* genotyped by *TaAP2* and *TaFAR* as markers showed a significant association with reduced height among elite varieties. It was present in about 76% of elite varieties indicating that it was positively selected in wheat breeding programs (Supplementary Table S3). In our previous study, *Rht24* reduced TGW by 6.9–8.5%. However, *Rht24* had a positive effect on thousand kernel weight in the present study (**Table 2**). We speculate that two reasons probably account for this case. The AK58/JD8 RIL and natural populations have better hereditary stability than the previous *F*_2:4_ mapping population and are used to define *Rht24* with more accurate effect analyses. Additionally, the interval of *Rht24* was narrowed down and thus more interference effects of other genes were excluded using the closest flanking markers, *TaFAR* and *TaAP2*. In fact, it still cannot be ruled out that other genes disturb the effect of *Rht24* on TGW based on our existing mapping information. Thus it is necessary to further narrow down the interval of *Rht24*. Thus, *Rht24* is an important QTL for plant height and TGW in wheat and MAS mediated by selection for appropriate *TaAP2* and *TaFAR* alleles should be effective.

**Table 3:** Phenotypic effect of *Rht24* on plant height in the AK58/JD8 RIL population.

Environment	Interval	LOD^a^	Add^b^	PVE (%)^c^
Anyang (2014–2015)	*TaAP2-TaFAR*	4.6	-3.1	9.9
Gaoyi (2014–2015)	*TaAP2-TaFAR*	11.7	-3.9	23.7
Gaoyi (2015–2016)	*TaAP2-TaFAR*	5.6	-3.1	10.7
Average	*TaAP2-TaFAR*	8.0	-3.4	16.8


*^a^Logarithm of odds score; QTLs were detected at a LOD threshold of 3.0. ^b^Additive effects. ^c^Percentage of phenotypic variance explained by *Rht24.**

Distribution of *Rht-B1b*, *Rht-D1b*, and *Rht8* in Chinese germplasm was detected by molecular markers, with frequencies of 24.5, 45.5, and 46.8%, respectively (Zhang et al., 2006). In addition, *Rht-B1b* and *Rht-D1b* were tested by KASP assays in current Chinese leading varieties with frequencies of 36.9 and 38.6%, respectively (Rasheed et al., 2016). Our study showed that *Rht24* was present in about 76% of elite Chinese varieties, a much higher frequency than either *Rht-B1b* or *Rht-D1b* (Supplementary Table S3). The results indicated that *Rht24* had the highest frequency distribution compared with the other three loci, and it was frequently present in combination with *Rht-D1b* or *Rht8*. It was reported that *Rht8* not only to reduce plant height but also significantly increase TGW (Zhang et al., 2016), however, *Rht-B1b* and *Rht-D1b* showed negative effects on TGW (Li et al., 1998). In this study, *Rht24* increased TGW by 2.0–3.4 g in all varieties used (**Table 2**).

In all, *Rht24* was important and extensively used in Chinese wheat breeding programs. Further study is needed to understand the origin of *Rht24* in Chinese wheat and investigate *Rht24* effect on the other traits, such as tiller number, flowering date, spike length and so on.

### Combination of SNP Chip-Based Screening and Genome Mining Was an Effective Approach for Fine Mapping of *Rht24*

As the development of genome sequencing technology, high-quality wheat genome sequencing data has been continuously updated and released^14^. Based on BLAST against wheat genome databases, the markers from preliminary mapping can be used as entry points to mine new markers linked to the loci of interest. In the present study we used SSR markers flanking *Rht24* as queries and identified a super scaffold. According to gene annotations, we developed three gene-specific markers and narrowed the region of *Rht24* from a 19.46 cM interval (8.2 cM in our previous study) to 1.8 cM. A 660K SNP chip was recently developed and released^15^. The 660K chip has several advantages compared to the 90K SNP chip, such as higher marker density and higher resolution as well as better distribution on chromosomes. We used this chip to screen candidate markers linked to *Rht24* and a significant number of polymorphic SNPs were obtained (data not shown). It was an obviously effective approach to take advantage of constantly updated genome information and high-throughput SNP platforms.

## Ethics Statement

We declare that these experiments comply with the ethical standards in China.

## Author Contributions

XT, LX, and DX performed the experiments. XT, WW, LF, CF, DW, XC, and QC did the field trials. SC and ZH designed the experiment. XT, SC, XX, and ZH wrote the paper.

## Conflict of Interest Statement

The authors declare that the research was conducted in the absence of any commercial or financial relationships that could be construed as a potential conflict of interest.

## Footnotes

1 http://wheat.pw.usda.gov

2 http://www.tw-biotech.com

3 http://tiangen.casmart.com.cn

4 http://www.transgen.com.cn

5 http://www.sangon.com/

6 http://plants.ensembl.org/Triticum_aestivum/Info/Index

7 http://www.lynnon.com

8 http://wheat.pw.usda.gov/ggpages/topics/Wheat660_SNP_array_developed_by_CAAS.pdf

9 http://www.capitalbiotech.com

10 http://www.isbreeding.net/

11 http://www.sas.com

12 http://wheat.pw.usda.gov

13 http://plants.ensembl.org/Triticum_aestivum/Info/Index

14 http://plants.ensembl.org/Triticum_aestivum

15 http://wheat.pw.usda.gov/ggpages/topics/Wheat660_SNP_array_developed_by_CAAS.pdf

## References

[B1] Asplund L. Leino M. W. Hagenblad J. (2012). Allelic variation at the *Rht8* locus in a 19th century wheat collection. *Sci. World J.* 2012 146–151. 10.1100/2012/385610 PMC3361164 22654600

[B3] Börner A. Schumann E. Fürste A. Cöster H. Leithold B. Röder S. (2002). Mapping of quantitative trait loci determining agronomic important characters in hexaploid wheat (*Triticum aestivum* L.). *Theor. Appl. Genet.* 105 921–936. 10.1007/s00122-002-0994-1 12582918

[B4] Buerstmayr H. Ban T. Anderson J. A. (2009). QTL mapping and marker-assisted selection for Fusarium head blight resistance in wheat: a review. *Plant Breed.* 128 1–26. 10.1111/j.1439-0523.2008.01550.x

[B6] Chen C. He Z. H. Lu J. L. Li J. Ren Y. Ma C. X. (2016). Molecular mapping of stripe rust resistance gene *YrJ22* in Chinese wheat cultivar Jimai 22. *Mol. Breed.* 36:118 10.1007/s11032-016-0540-5

[B7] Chen S. L. Gao R. H. Wang H. Y. Wen M. X. Xiao J. Bian N. F. (2015). Characterization of a novel reduced height gene (*Rht23*) regulating panicle morphology and plant architecture in bread wheat. *Euphytica* 203 583–594. 10.1007/s10681-014-1275-1

[B8] Colasuonno P. Gadaleta A. Giancaspro A. Nigro D. Giove S. Incerti O. (2014). Development of a high-density SNP-based linkage map and detection of yellow pigment content QTLs in durum wheat. *Mol. Breed.* 34 1563–1578. 10.1007/s11032-014-0183-3

[B10] Ellis M. Spielmeyer W. Gale K. Rebetzke G. Richards R. (2002). “Perfect” markers for the *Rht-B1b* and *Rht-D1b* dwarfing genes in wheat. *Theor. Appl. Genet.* 105 1038–1042. 10.1007/s00122-002-1048-4 12582931

[B11] Ellis M. H. Rebetzke G. J. Azanza F. Richards R. A. Spielmeyer W. (2005). Molecular mapping of gibberellin-responsive dwarfing genes in bread wheat. *Theor. Appl. Genet.* 111 423–430. 10.1007/s00122-005-2008-6 15968526

[B12] Evans L. T. (1998). Crop evolution, adaptation and yield. *Photosynthetica* 34 56–60. 10.1023/A:1006889901899

[B13] Gale M. D. Law C. N. (1973). Semi-dwarf wheats induced by monosomy and associated changes in gibberellin levels. *Nature* 241 211–212. 10.1038/241211a0

[B14] Gao L. Turner M. K. Chao S. Kolmer J. Anderson J. A. (2016). Genome wide association study of seedling and adult plant leaf rust resistance in elite spring wheat breeding lines. *PLoS ONE* 11:e0148671 10.1371/journal.pone.0148671 PMC4744023 26849364

[B15] Griffiths S. Simmonds J. Leverington M. Wang Y. Fish L. J. Sayers L. (2012). Meta-QTL analysis of the genetic control of crop height in elite European winter wheat germplasm. *Mol. Breed.* 29 159–171. 10.1007/s00122-009-1046-x 19430758

[B17] Jin H. Wen W. E. Liu J. D. Zhai S. N. Zhang Y. Yan J. (2016). Genome-wide QTL mapping for wheat processing quality parameters in a Gaocheng 8901/Zhoumai 16 recombinant inbred line population. *Front. Plant Sci.* 7:1032 10.1007/s00122-006-0346-7 PMC4949415 27486464

[B19] Korzun V. Röder M. S. Ganal M. W. Worland A. J. Law C. N. (1998). Genetic analysis of the dwarfing gene (*Rht8*) in wheat. Part I. Molecular mapping of *Rht8* on the short arm of chromosome 2D of bread wheat (*Triticum aestivum* L.). *Theor. Appl. Genet.* 96 1104–1109. 10.1007/s001220050845

[B20] Law C. N. Snape J. W. Worland A. J. (1978). The genetic relationship between height and yield in wheat. *Heredity* 40 15–20. 10.1038/hdy.1978.13

[B21] Li X. M. Xia X. C. Xiao Y. G. He Z. H. Wang D. S. Trethowan R. (2015). QTL mapping for plant height and yield components in common wheat under water-limited and full irrigation environments. *Crop Pasture Sci.* 66 660–670. 10.1071/CP14236

[B22] Li X. P. Jiang C. Z. Liu H. L. (1998). Effects of different dwarfing genes on agronomic characteristics of winter wheat. *Acta Agron. Sin.* 24 475–478.

[B23] Lincoln S. E. Daly M. J. Lander E. S. (1992). *Constructing Genetic Maps with MapMaker/EXP3.0*, 3rd Edn Cambridge, MA: Whitehead Institute.

[B24] Liu G. Xu S. B. Ni Z. F. Xie C. J. Qin D. D. Li J. (2011). Molecular dissection of plant height QTLs using recombinant inbred lines from hybrids between common wheat (*Triticum aestivum* L.) and spelt wheat *(Triticum spelta L.)*. *Chin. Sci. Bull.* 56 1897–1903. 10.1007/s11434-011-4506-z

[B25] Liu J. D. He Z. H. Wu L. Bai B. Wen W. E. Xie C. J. (2016). Genome-wide linkage mapping of QTL for black point reaction in bread wheat (*Triticum aestivum* L.). *Theor. Appl. Genet.* 129 2179–2190. 10.1007/s00122-016-2766-3 27531362

[B26] Mccartney C. A. Somers D. J. Humphreys D. G. Lukow O. Ames N. Noll J. S. (2005). Mapping quantitative trait loci controlling agronomic traits in the spring wheat cross RL4452 × ‘AC Domain’. *Genome* 48 870–883. 10.1139/g05-055 16391693

[B27] McIntosh R. A. Dubcovsky J. Rogers W. J. Morris C. Appels R. Xia X. C. (2015). *Catalogue of Gene Symbols for Wheat: 2015–2016. Supplement.* Available at: http://shigen.nig.ac.jp/wheat/komugi/genes/macgene/supplement2015.pdf

[B28] McIntosh R. A. Hart G. E. Gale M. D. (2013). *Catalogue of Gene Symbols for Wheat: 2013-2014 Supplement.* Available at: http://shigen.nig.ac.jp/wheat/komugi/genes/macgene/supplement2013-2014.pdf

[B29] Mir R. R. Kumar N. Jaiswal V. Girdharwal N. Prasad M. Balyan H. S. (2012). Genetic dissection of grain weight in bread wheat through quantitative trait locus interval and association mapping. *Mol. Breed.* 29 963–972. 10.1007/s11032-011-9693-4

[B30] Naruoka Y. Garland-Campbell K. A. Carter A. H. (2015). Genome-wide association mapping for stripe rust (Puccinia striiformis f. sp. *tritici*) in US Pacific Northwest winter wheat (*Triticum aestivum* L.). *Theor. Appl. Genet.* 128 1083–1101. 10.1007/s00122-015-2492-2 25754424

[B31] Nyquist W. E. Baker R. J. (1991). Estimation of heritability and prediction of selection response in plant populations. *Crit. Rev. Plant Sci.* 10 235–322. 10.1080/07352689109382313

[B32] Pearce S. Saville R. Vaughan S. P. Chandler P. M. Wilhelm E. P. Alkaff N. (2011). Molecular characterization of *Rht-1* dwarfing genes in hexaploid wheat. *Plant Physiol.* 157 1820–1831. 10.1104/pp.111.183657 22013218 PMC3327217

[B33] Peng J. Richards D. E. Hartley N. M. Murphy G. P. Devos K. M. Flintham J. E. (1999). ‘Green revolution’ genes encode mutant gibberellin response modulators. *Nature* 400 256–261. 10.1038/22307 10421366

[B34] Peng J. H. Ronin Y. Fahima T. (2003). Domestication quantitative trait loci in Triticum dicoccoides, the progenitor of wheat. *Proc. Natl. Acad. Sci. U.S.A.* 100 2489–2494. 10.1073/pnas.252763199 12604784 PMC151368

[B35] Peng Z. S. Li X. Yang Z. J. Liao M. L. (2011). A new reduced height gene found in the tetraploid semi-dwarf wheat landrace Aiganfanmai. *Genet. Mol. Res.* 10 2349–2357. 10.4238/2011 22002128

[B36] Quarrie S. A. Steed A. Calestani C. (2005). High-density genetic map of hexaploid wheat (*Triticum aestivum* L.) from the cross Chinese Spring x SQ1 and its use to compare QTLs for grain yield across a range of environments. *Theor. Appl. Genet.* 110 865–880. 10.1007/s00122-004-1902-7 15719212

[B37] Rasheed A. Wen W. E. Gao F. M. Zhai S. N. Jin H. Liu J. D. (2016). Development and validation of KASP assays for genes underpinning key economic traits in bread wheat. *Theor. Appl. Genet.* 129 1843–1860. 10.1007/s00122-016-2743-x 27306516

[B38] Rebetzke G. J. Richards R. A. (2000). Gibberellic acid-sensitive dwarfing genes reduce plant height to increase kernel number and grain yield of wheat. *Crop Pasture Sci.* 51 235–246. 10.1071/AR99043

[B39] Röder M. S. Korzun V. Wendehake K. Plaschke J. Tixier M. H. Leroy P. (1998). A microsatellite map of wheat. *Genetics* 149 2007–2023. 9691054 10.1093/genetics/149.4.2007 PMC1460256

[B40] Rogers J. (2014). “The IWGSC survey sequencing initiative,” in *Proceedings of the International Plant and Animal Genome Conference XXII*, San Diego, CA.

[B41] Saghai-Maroof M. A. Soliman K. M. Jorgensen R. A. Allard R. W. (1984). Ribosomal DNA spacer-length polymorphisms in barley: mendelian inheritance, chromosomal location, and population dynamics. *Proc. Natl. Acad. Sci. U.S.A.* 24 8014–8018. 10.1073/pnas.81.24.8014 PMC392284 6096873

[B42] Sukumaran S. Dreisigacker S. Lopes M. Chavez P. Reynolds M. P. (2015). Genome-wide association study for grain yield and related traits in an elite spring wheat population grown in temperate irrigated environments. *Theor. Appl. Genet.* 128 353–363. 10.1007/s00122-014-2435-3 25490985

[B43] Thiel T. Kota R. Grosse I. Stein N. Graner A. (2004). SNP2CAPS: a SNP and indel analysis tool for CAPS marker development. *Nucl. Acids Res.* 32:e5 10.1093/nar/gnh006 PMC373308 14704362

[B44] Wang S. C. Wong D. Forrest K. Allen A. L. Chao S. Huang B. E. (2014). Characterization of polyploid wheat genomic diversity using the high-density 90,000 SNP array. *Plant Biotechnol. J.* 12 787–796. 10.1111/pbi.12183 24646323 PMC4265271

[B45] Wei T. M. Chang X. P. Min D. H. Jing R. L. (2010). Analysis of genetic diversity and tapping elite alleles for plant height in drought-tolerant wheat varieties. *Acta Agron. Sin.* 36 895–904. 10.3724/SP.J.1006.2010.00895

[B46] Worland A. J. Korzun V. Röder M. S. Ganal M. W. Law C. N. (1998). Genetic analysis of the dwarfing gene *Rht8* in wheat. Part II. The distribution and adaptive significance of allelic variants at the *Rht8* locus of wheat as revealed by microsatellite screening. *Theor. Appl. Genet.* 96 1110–1120. 10.1007/s001220050846

[B47] Wu J. Kong X. Wan J. Liu X. Zhang X. Guo X. (2011). Dominant and pleiotropic effects of a *GAI* gene in wheat results from a lack of interaction between DELLA and GID1. *Plant Physiol.* 157 2120–2130. 10.1104/pp.111.185272 22010107 PMC3327208

[B48] Wu Q. H. Chen Y. G. Fu L. Zhou S. H. Chen J. J. Zhao X. J. (2016). QTL mapping of flag leaf traits in common wheat using an integrated high-density SSR and SNP genetic linkage map. *Euphytica* 208 337–351. 10.1007/s10681-015-1603-0

[B49] Würschum T. Langer S. M. Longin C. F. (2015). Genetic control of plant height in European winter wheat cultivars. *Theor. Appl. Genet.* 128 865–874. 10.1007/s00122-015-2476-2 25687129

[B51] Zhai S. N. He Z. H. Wen W. E. Jin H. Liu J. D. Zhang Y. (2016). Genome-wide linkage mapping of flour color-related traits and polyphenol oxidase activity in common wheat. *Theor. Appl. Genet.* 129 377–394. 10.1007/s00122-015-2634-6 26602234

[B52] Zhang D. Q. Song X. P. Feng J. Ma W. J. Wu B. J. Zhang C. L. (2016). Detection of dwarf genes *Rht-B1b*, *Rht-D1b* and *Rht8* in huang-huai valley winter wheat areas and the influence on agronomic characteristics. *J. Triticeae Crops* 36 975–981. 10.7606/j.ssn.1009-1041.2016.08.01

[B53] Zhang X. K. Yang S. J. Zhou Y. He Z. H. Xia X. C. (2006). Distribution of the *Rht-B1b*, *Rht-D1b* and *Rht8* reduced height genes in autumn-sown Chinese wheats detected by molecular markers. *Euphytica* 152 109–116. 10.1007/s10681-006-9184-6

