# Supplementary material for: Molecular Mapping of Reduced Plant Height Gene Rht24 in Bread Wheat
Source: Front Plant Sci. 2017 Aug 8;8:1379. doi: 10.3389/fpls.2017.01379 (PMC5550838; doi:10.3389/fpls.2017.01379)
Supplement: Supplementary file 1 [file Table_1.docx]

**Supplementary Table S1** The main dwarfing genes in wheat

| New name | Old name | Position | Cloned | Reference |
| --- | --- | --- | --- | --- |
| *Rht-B1b* | *Rht1* | 4BS | Yes | Peng et al., 1999 |
| *Rht-B1c* | *Rht3* | 4BS | Yes | Pearce et al., 2011 |
| *Rht-D1b* | *Rht2* | 4DS | Yes | Peng et al., 1999 |
| *Rht-D1c* | *Rht10* | 4DS | Yes | Pearce et al., 2011 |
| *Rht4* | *Rht4* | 2BL | No | Ellis et al., 2005 |
| *Rht5* | *Rht5* | 3BS | No | Daoura. et al., 2013 |
| *Rht6* | *Rht6* |  | No | Konzak, 1976 |
| *Rht7* | *Rht7* | 2AS | No | Chaudhry, 1973 |
| *Rht8* | *Rht8* | 2DS | No | Worland et al., 1998 |
| *Rht9* | *Rht9* | 7BS | No | Law et al., 1978 |
| *Rht11* | *Rht11* |  | No | Ellis et al., 2005 |
| *Rht12* | *Rht12* | 5AL | No | Ellis et al., 2005 |
| *Rht13* | *Rht13* | 7BS | No | Ellis et al., 2005 |
| *Rht14* | *Rht14* | 6AS | No | Haque et al ., 2011 |
| *Rht15* | *Rht15* |  | No | Konzak, 1976 |
| *Rht16* | *Rht16* | 6AS | No | Haque et al ., 2011 |
| *Rht17* | *Rht17* |  | No | Bazhenov et al., 2015 |
| *Rht18* | *Rht18* | 6AS | No | Haque et al ., 2011 |
| *Rht19* | *Rht19* |  | No | Konzak, 1976 |
| *Rht20* | *Rht20* |  | No | Konzak, 1976 |
| *Rht21* | *Rht21* | 2AS | No | Yang et al., 1993 |
| *Rht22* | *Rht22* | 7AS | No | Peng et al., 2011 |
| *Rht23* | *Rht23* | 5DL | No | Chen et al., 2015 |

**Reference:**

Bazhenov, M. S., Divashuk, M. G., Amagai, Y., Watanabe, N., and Karlov, G. I. (2015). Isolation of the dwarfing Rht-B1p (*Rht17*) gene from wheat and the development of an allele-specific PCR marker. *Mol. Breed.* 35:213. doi: 10.1007/ s11032-015-0407-1

Chaudhry, A. (1973). *A Genetic and Cytogenetic Study of Height in Wheat*. Doctoral dissertation, University of Cambridge, Cambridge.

Chen, S. L., Gao, R. H., Wang, H. Y., Wen, M. X., Xiao, J., Bian, N. F., et al. (2015). Characterization of a novel reduced height gene (*Rht23*) regulating panicle morphology and plant architecture in bread wheat. *Euphytica* 203, 583–594. doi: 10.1007/s10681-014-1275-1

Daoura, B. G., Liang, C., and Hu, Y. G. (2013). Agronomic traits affected by dwarfing gene *Rht5* in common wheat (*Triticum aestivum* L.). *Aust. J. Crop Sci.* 7, 1270–1276.

Ellis, M. H., Rebetzke, G. J., Azanza, F., Richards, R. A., and Spielmeyer, W. (2005). Molecular mapping of gibberellin-responsive dwarfing genes in bread wheat. *Theor. Appl. Genet.* 111, 423–430. doi: 10.1007/s00122-005-2008-6 Haque, M. A., Martinek, P., Watanabe, N., and Kuboyama, T. (2011). Genetic mapping of gibberellic acid-sensitive genes for semi-dwarfism in durum wheat. Cereal Res. Commun. 39, 171–178. doi: 10.1556/CRC.39.2011.2.1

Konzak, C. F. (1976). *A Review of Semid-Warfing Gene Sources, and a Description of Some New Mutants Useful for Breeding Short-Stature Wheats*. Vienna: Induced Mutations in Cross-Breeding.

Law, C. N., Snape, J. W., and Worland, A. J. (1978). The genetic relationship between height and yield in wheat. *Heredity* 40, 15–20. doi:10.1038/hdy.1978.13 Pearce, S., Saville, R., Vaughan, S. P., Chandler, P. M., Wilhelm, E. P., Alkaff, N., et al. (2011). Molecular characterization of Rht-1 dwarfing genes in hexaploid wheat. *Plant Physiol.* 157, 1820–1831. doi: 10.1104/pp.111.183657

Peng, J., Richards, D. E., Hartley, N. M., Murphy, G. P., Devos, K. M., Flintham, J. E., et al. (1999). ‘Green revolution’ genes encode mutant gibberellin response modulators. *Nature* 400, 256–261. doi: 10.1038/22307

Peng, Z. S., Li, X., Yang, Z. J., and Liao, M. L. (2011). A new reduced height gene found in the tetraploid semi-dwarf wheat landrace Aiganfanmai. *Genet. Mol.Res.* 10, 2349–2357. doi: 10.4238/2011

Worland, A. J., Korzun, V., Röder, M. S., Ganal, M. W., and Law, C. N. (1998). Genetic analysis of the dwarfing gene Rht8 in wheat. Part II. The distribution and adaptive significance of allelic variants at the Rht8 locus of wheat as revealed by microsatellite screening. *Theor. Appl. Genet.* 96, 1110–1120. doi: 10.1007/ s001220050846

Yang, T. Z., Zhang, X. K., Liu, H. W., and Wang, Z. H. (1993). Chromosomal arm location of a dominant dwarfing gene Rht21 in common wheat variety—XN0004. *J. Northwest A F Univ.* 12, 13–17.
