# Supplementary material for: Molecular Mapping of Reduced Plant Height Gene Rht24 in Bread Wheat
Source: Front Plant Sci. 2017 Aug 8;8:1379. doi: 10.3389/fpls.2017.01379 (PMC5550838; doi:10.3389/fpls.2017.01379)
Supplement: Supplementary file 2 [file Table_2.DOCX]

**Supplementary Table 2** Markers linked to the *Rht24*

| Marker | Primer name | Primer sequence (5ˊ–3ˊ) | PCR fragment (bp) |
| --- | --- | --- | --- |
| *TaGA3* | *TaGA3*-F | GTGGGGCCGTTCACGGGATAC | 1007 |
|  | *TaGA3*-R | AGAGTGGTGAGGCGAAGTGAAATTGATTC |  |
|  | *TaGA3*-CAPS-F | TAGAATGATGGCTCACTGCTGAGGCTACGA | 848 |
| *TaFAR* | *TaFAR*-F | CCCATACTACTCTGGAACTTGCCCATCTC | 817 |
|  | *TaFAR*-R | GATCGGTTAAGAAAAATCATTTACATGCCCTCAAC |  |
|  | *TaFAR*-CAPS-R | ATATTTACCAACTCCTGGGATGTCCCAACA | 539 |
| *TaAP2* | *TaAP2*-F | GGTTAGAACATTATATTATTAATTACTGGTTGATTTCTTATATTG | 488 |
|  | *TaAP2*-R | CTCGTATGAACAAGTAATTCAATTCAATTATAAATAAGTC |  |
| *TaSNP1* | *TaSNP1*-F | CTAAACTTCATTTCTGCAATTTTGAAT | 573 |
|  | *TaSNP1*-R | GAAACCAAATGAATTTCAGTTACG |  |
| *TaSNP2* | *TaSNP2*-F | TTGCCCTGGAACACAGTCTGATG | 389 |
|  | *TaSNP2*-R | CGCGATTTGGACTGCAGTGAAT |  |
| *TaSNP3* | *TaSNP3*-F | GCCAACTGCGAATTCCTTGCA | 637 |
|  | *TaSNP3*-R | CACCCAGAACGTACCCAGAGGC |  |
