# Supplementary material for: Molecular Mapping of Reduced Plant Height Gene Rht24 in Bread Wheat
Source: Front Plant Sci. 2017 Aug 8;8:1379. doi: 10.3389/fpls.2017.01379 (PMC5550838; doi:10.3389/fpls.2017.01379)
Supplement: Supplementary file 3 [file Table_3.DOCX]

**Supplementary Table 3** Frequencies of genotypes among 242 Chinese and introduced wheat varieties

| Genotype^a^ | No. of introduced varieties | No. of Chinese varieties | Total | Frequency (%) |
| --- | --- | --- | --- | --- |
| *TaFAR-****a/****TaAP2-****a*** | 23 | 18 | 41 | 17.0 |
| *TaFAR-****a/****TaAP2-****b*** | 1 | 6 | 7 | 2.9 |
| *TaFAR-****b/****TaAP2-****a*** | 3 | 6 | 9 | 3.7 |
| *TaFAR-****b/****TaAP2-****b*** | 37 | 148 | 185 | 76.4 |

^a^ *FAR-****a***/*AP2-****a***, JD8 parental type; *FAR-****b***/*AP2-****b***, AK58 parental type; *FAR-****a***/*AP2-****b*** and *FAR-****b***/*AP2-****a***, recombinant types
