# Supplementary material for: Molecular Mapping of Reduced Plant Height Gene Rht24 in Bread Wheat
Source: Front Plant Sci. 2017 Aug 8;8:1379. doi: 10.3389/fpls.2017.01379 (PMC5550838; doi:10.3389/fpls.2017.01379)
Supplement: Supplementary file 4 [file Table_4.DOCX]

**Supplementary Table** **4** *Rht24* genotypes and plant height of wheat varieties in Set I germplasm

| Code | Variety | Origin | Genotype^a^ | Plant height (cm) | | | |
| --- | --- | --- | --- | --- | --- | --- | --- |
|  |  |  |  | 2012–2013 Anyang | 2012–2013 Suixi | 2013–2014 Anyang | 2013–2014 Suixi |
| 1 | An 1331 | Anhui | B | 85.0 | 86.0 | 83.9 | 87.2 |
| 2 | Huaimai 18 | Anhui | B | 75.7 | 71.7 | 70.6 | 75.7 |
| 3 | Huaimai 20 | Anhui | B | 78.3 | 76.3 | 74.0 | 80.5 |
| 4 | Huaimai 21 | Anhui | B | 80.7 | 74.3 | 78.4 | 82.3 |
| 5 | Su 0663 | Anhui | B | 75.7 | 72.0 | 73.1 | 76.1 |
| 6 | Wan 23094 | Anhui | A | 84.0 | 81.0 | 76.2 | 81.3 |
| 7 | Wanmai 19 | Anhui | B | 90.0 | 87.0 | 87.1 | 86.4 |
| 8 | Wanmai 29 | Anhui | B | 84.3 | 85.0 | 82.6 | 84.3 |
| 9 | Wanmai 33 | Anhui | B | 77.3 | 72.3 | 63.0 | 78.1 |
| 10 | Wanmai 38 | Anhui | B | 76.7 | 74.3 | 74.4 | 78.2 |
| 11 | Wanmai 50 | Anhui | B | 80.0 | 78.3 | 75.9 | 76.1 |
| 12 | Wanmai 52 | Anhui | B | 81.7 | 78.3 | 80.3 | 83.7 |
| 13 | Wanmai 53 | Anhui | B | 77.3 | 76.7 | 73.7 | 76.7 |
| 14 | Dan 6172 | Hebei | B | 80.7 | 77.7 | 73.7 | 79.0 |
| 15 | Fu 936 | Hebei | B | 74.3 | 70.0 | 69.8 | 76.5 |
| 16 | Gaocheng 8901 | Hebei | B | 90.0 | 95.0 | 87.9 | 93.6 |
| 17 | Gaoyou 503 | Hebei | D | 95.0 | 93.3 | 79.8 | 95.9 |
| 18 | Guan 35 | Hebei | B | 75.0 | 73.7 | 68.9 | 73.6 |
| 19 | Heng 7228 | Hebei | B | 81.7 | 79.0 | 74.2 | 83.2 |
| 20 | Hengguan 33 | Hebei | B | 80.0 | 74.3 | 71.9 | 82.2 |
| 21 | Jinhe 9123 | Hebei | B | 76.7 | 80.0 | 75.6 | 78.7 |
| 22 | Jishi 02-1 | Hebei | B | 78.0 | 76.7 | 74.3 | 79.0 |
| 23 | Shi 4185 | Hebei | A | 83.3 | 78.3 | 74.8 | 81.1 |
| 24 | Shijiazhuang 15 | Hebei | D | 82.3 | 80.3 | 74.2 | 78.3 |
| 25 | Shijiazhuang 8 | Hebei | B | 85.0 | 81.0 | 78.9 | 82.0 |
| 26 | Shixin 733 | Hebei | B | 86.3 | 81.3 | 78.1 | 84.4 |
| 27 | Shixin 828 | Hebei | B | 84.0 | 82.3 | 73.5 | 81.1 |
| 28 | Shiyou 17 | Hebei | B | 83.3 | 79.3 | 77.0 | 76.9 |
| 29 | 11CA40 | Henan | B | 70.0 | 63.7 | 59.5 | 66.1 |
| 30 | 85 zhong 33 | Henan | B | 82.3 | 74.0 | 73.7 | 82.1 |
| 31 | Aikang 58 | Henan | B | 64.3 | 62.3 | 61.2 | 64.9 |
| 32 | Bainong 3217 | Henan | C | 83.0 | 75.3 | 76.8 | 80.2 |
| 33 | Bainong 64 | Henan | B | 82.7 | 77.0 | 80.4 | 79.3 |
| 34 | Huapei 5 | Henan | A | 79.3 | 71.0 | 72.7 | 76.4 |
| 35 | Lankao 2 | Henan | B | 75.0 | 68.7 | 74.4 | 77.9 |
| 36 | Lankao 24 | Henan | D | 73.3 | 67.0 | 69.1 | 73.8 |
| 37 | Lankao 906 | Henan | B | 80.7 | 70.3 | 74.4 | 81.6 |
| 38 | Luohan 2 | Henan | B | 84.0 | 80.7 | 79.1 | 80.8 |
| 39 | Luomai 21 | Henan | B | 83.3 | 81.7 | 78.2 | 80.9 |
| 40 | Neixiang 188 | Henan | B | 75.0 | 71.3 | 65.3 | 74.6 |
| 41 | Neixiang 5 | Henan | A | 138.3 | 125.0 | 135.7 | 125.8 |
| 42 | Xinmai 19 | Henan | B | 75.0 | 73.3 | 69.9 | 76.3 |
| 43 | Xinmai 9 | Henan | B | 78.3 | 78.7 | 73.2 | 76.7 |
| 44 | Xinmai 9408 | Henan | B | 76.7 | 75.3 | 71.2 | 73.0 |
| 45 | Yanzhan 4110 | Henan | B | 75.0 | 73.3 | 75.9 | 78.6 |
| 46 | Yumai 13 | Henan | B | 83.3 | 75.3 | 72.4 | 77.4 |
| 47 | Yumai 18 | Henan | B | 77.3 | 77.0 | 72.0 | 82.3 |
| 48 | Yumai 2 | Henan | B | 90.0 | 80.3 | 78.6 | 85.0 |
| 49 | Yumai 21 | Henan | B | 81.7 | 72.0 | 67.3 | 76.1 |
| 50 | Yumai 34 | Henan | B | 80.0 | 79.3 | 66.0 | 78.1 |
| 51 | Yumai 35 | Henan | B | 76.0 | 73.7 | 62.1 | 71.0 |
| 52 | Yumai 47 | Henan | B | 80.0 | 75.3 | 73.8 | 75.9 |
| 53 | Yumai 49 | Henan | B | 75.0 | 74.7 | 70.0 | 73.0 |
| 54 | Yumai 50 | Henan | B | 93.3 | 88.7 | 85.6 | 93.1 |
| 55 | Yumai 57 | Henan | B | 77.3 | 76.3 | 69.5 | 76.9 |
| 56 | Yumai 63 | Henan | B | 81.7 | 79.0 | 63.8 | 79.5 |
| 57 | Yumai 7 | Henan | A | 75.0 | 75.3 | 66.0 | 72.5 |
| 58 | Zheng 9023 | Henan | B | 84.3 | 80.3 | 73.8 | 83.5 |
| 59 | Zhengmai 366 | Henan | B | 65.0 | 55.0 | 58.2 | 59.8 |
| 60 | Zhong 892 | Henan | B | 76.3 | 75.7 | 64.6 | 74.9 |
| 61 | Zhongmai 871 | Henan | B | 75.0 | 75.0 | 68.6 | 76.0 |
| 62 | Zhongmai 875 | Henan | B | 73.7 | 71.7 | 71.1 | 75.1 |
| 63 | Zhongmai 895 | Henan | B | 70.0 | 67.3 | 62.3 | 67.2 |
| 64 | Zhongyu 9 | Henan | D | 80.3 | 82.7 | 72.8 | 81.0 |
| 65 | Zhou 8425 B | Henan | B | 70.0 | 68.7 | 67.9 | 71.1 |
| 66 | Zhoumai 11 | Henan | B | 76.7 | 74.0 | 67.8 | 75.5 |
| 67 | Zhoumai 12 | Henan | B | 81.3 | 76.0 | 77.8 | 82.7 |
| 68 | Zhoumai 13 | Henan | B | 63.3 | 59.0 | 59.0 | 63.4 |
| 69 | Zhoumai 16 | Henan | B | 68.3 | 68.3 | 62.9 | 68.6 |
| 70 | Zhoumai 18 | Henan | B | 75.3 | 75.3 | 71.1 | 72.9 |
| 71 | Zhoumai 19 | Henan | B | 78.0 | 73.7 | 72.6 | 77.2 |
| 72 | Zhoumai 22 | Henan | B | 72.3 | 73.3 | 70.9 | 74.5 |
| 73 | Zhoumai 23 | Henan | B | 78.3 | 76.7 | 75.9 | 78.5 |
| 74 | Zhoumai 25 | Henan | B | 76.7 | 72.7 | 70.0 | 77.8 |
| 75 | Zhoumai 26 | Henan | B | 75.0 | 74.0 | 72.2 | 75.7 |
| 76 | Zhoumai 28 | Henan | B | 74.0 | 76.0 | 73.4 | 77.6 |
| 77 | Zhoumai 30 | Henan | B | 75.0 | 74.0 | 69.2 | 74.7 |
| 78 | Zhoumai 31 | Henan | B | 80.0 | 73.3 | 74.2 | 78.9 |
| 79 | Zhoumai 32 | Henan | B | 73.3 | 73.3 | 64.4 | 72.3 |
| 80 | Aifeng 3 | Shaanxi | B | 92.3 | 83.7 | 86.6 | 90.8 |
| 81 | Shaan 150 | Shaanxi | B | 91.7 | 84.7 | 89.0 | 89.6 |
| 82 | Shaan 229 | Shaanxi | C | 88.3 | 84.0 | 82.7 | 89.2 |
| 83 | Shaan 253 | Shaanxi | B | 81.7 | 81.7 | 64.5 | 81.0 |
| 84 | Shaan 354 | Shaanxi | B | 80.7 | 79.7 | 72.2 | 78.1 |
| 85 | Shaan 715 | Shaanxi | B | 87.3 | 81.3 | 75.0 | 84.3 |
| 86 | Shaan 512 | Shaanxi | B | 86.7 | 82.0 | 78.0 | 89.2 |
| 87 | Shaanmai 509 | Shaanxi | B | 76.3 | 74.7 | 68.8 | 76.4 |
| 88 | Shaanmai 94 | Shaanxi | B | 76.7 | 71.7 | 69.7 | 76.1 |
| 89 | Shaannong 78-59 | Shaanxi | B | 86.0 | 86.7 | 80.6 | 85.4 |
| 90 | Shaannong 981 | Shaanxi | B | 87.7 | 85.7 | 81.2 | 81.9 |
| 91 | Shaanyou 225 | Shaanxi | B | 89.0 | 83.7 | 79.7 | 83.4 |
| 92 | Wunong 148 | Shaanxi | B | 85.7 | 81.7 | 73.6 | 83.9 |
| 93 | Xiaoyan 22 | Shaanxi | B | 84.3 | 84.3 | 74.9 | 83.2 |
| 94 | Xiaoyan 54 | Shaanxi | B | 91.0 | 84.7 | 83.7 | 92.1 |
| 95 | Xiaoyan 6 | Shaanxi | D | 90.7 | 94.0 | 89.4 | 91.0 |
| 96 | Xiaoyan 81 | Shaanxi | B | 84.3 | 77.7 | 76.5 | 83.8 |
| 97 | Xinong 1376 | Shaanxi | B | 74.3 | 67.3 | 59.3 | 75.0 |
| 98 | Xinong 2000-7 | Shaanxi | B | 73.3 | 72.0 | 68.4 | 73.9 |
| 99 | Xinong 291 | Shaanxi | B | 85.3 | 80.3 | 82.1 | 91.6 |
| 100 | Xinong 88 | Shaanxi | A | 90.0 | 82.0 | 83.2 | 84.5 |
| 101 | Xinong 979-005 | Shaanxi | B | 73.3 | 71.7 | 64.0 | 74.5 |
| 102 | Jimai 19 | Shandong | B | 85.0 | 75.0 | 73.9 | 80.2 |
| 103 | Jimai 20 | Shandong | B | 83.3 | 79.0 | 70.6 | 76.9 |
| 104 | Jimai 21 | Shandong | C | 74.0 | 70.0 | 70.3 | 70.2 |
| 105 | Jimai 22 | Shandong | B | 75.0 | 75.7 | 66.8 | 73.8 |
| 106 | Jinan 13 | Shandong | B | 91.7 | 88.3 | 85.8 | 91.3 |
| 107 | Jinan 17 | Shandong | B | 80.0 | 79.7 | 75.8 | 80.8 |
| 108 | Jining 16 | Shandong | B | 78.3 | 65.3 | 71.1 | 85.0 |
| 109 | Lingxing 66 | Shandong | B | 80.0 | 71.3 | 71.9 | 73.6 |
| 110 | Lingxing 99 | Shandong | B | 82.3 | 76.0 | 74.7 | 80.4 |
| 111 | Linmai 2 | Shandong | B | 83.3 | 81.0 | 82.0 | 92.0 |
| 112 | Linmai 4 | Shandong | B | 80.0 | 67.7 | 70.5 | 82.2 |
| 113 | Lumai 11 | Shandong | A | 90.0 | 84.3 | 81.4 | 84.9 |
| 114 | Lumai 14 | Shandong | B | 80.0 | 75.3 | 74.4 | 80.7 |
| 115 | Lumai 15 | Shandong | B | 84.3 | 80.7 | 82.9 | 85.5 |
| 116 | Lumai 21 | Shandong | B | 83.3 | 80.0 | 78.7 | 87.4 |
| 117 | Lumai 23 | Shandong | B | 87.3 | 76.0 | 77.2 | 86.5 |
| 118 | Lumai 5 | Shandong | C | 83.7 | 77.7 | 75.3 | 79.9 |
| 119 | Lumai 6 | Shandong | B | 90.0 | 85.7 | 81.2 | 86.7 |
| 120 | Lumai 7 | Shandong | A | 76.0 | 72.0 | 75.2 | 76.7 |
| 121 | Lumai 8 | Shandong | B | 77.0 | 73.3 | 69.1 | 75.3 |
| 122 | Lumai 9 | Shandong | B | 90.0 | 84.0 | 79.0 | 85.5 |
| 123 | Luyuan 502 | Shandong | B | 81.7 | 74.3 | 73.0 | 78.9 |
| 124 | PH82-2 | Shandong | B | 90.0 | 85.3 | 80.2 | 88.0 |
| 125 | Shannong 20 | Shandong | B | 80.0 | 79.0 | 72.7 | 81.6 |
| 126 | Taishan 1 | Shandong | B | 106.7 | 95.7 | 92.0 | 100.9 |
| 127 | Taishan 5 | Shandong | B | 90.7 | 84.3 | 80.9 | 87.6 |
| 128 | Yannong 15 | Shandong | B | 84.0 | 73.7 | 73.9 | 84.6 |
| 129 | Yannong 18 | Shandong | A | 91.0 | 86.3 | 87.4 | 89.1 |
| 130 | Yannong 19 | Shandong | B | 81.7 | 83.3 | 78.6 | 82.4 |
| 131 | Zimai 12 | Shandong | B | 83.7 | 77.7 | 78.2 | 83.3 |
| 132 | Zixuan 2 | Shandong | B | 85.7 | 83.0 | 81.7 | 89.0 |
| 133 | Wennong 14 | Shandong | B | 77.7 | 76.0 | 73.6 | 77.4 |
| 134 | Wennong 5 | Shandong | B | 70.0 | 68.0 | 70.3 | 75.1 |
| 135 | Jinmai 61 | Shanxi | B | 87.3 | 79.0 | 82.3 | 81.7 |
| 136 | Linhan 2 | Shanxi | B | 93.3 | 84.0 | 85.6 | 88.0 |
| 137 | Linkang 12 | Shanxi | B | 95.7 | 83.7 | 86.2 | 89.4 |
| 138 | Aca 601 | Argentina | A | 80.0 | 79.3 | 79.6 | 85.2 |
| 139 | Aca 801 | Argentina | B | 95.0 | 88.3 | 92.1 | 92.1 |
| 140 | Klein Jabal 1 | Argentina | A | 103.3 | 95.7 | 95.0 | 100.8 |
| 141 | Nidera Baguette 10 | Argentina | A | 88.3 | 89.3 | 88.1 | 97.1 |
| 142 | Nidera Baguette 20 | Argentina | B | 81.0 | 83.7 | 75.5 | 82.2 |
| 143 | Barra | Italy | B | 81.7 | 77.3 | 73.0 | 79.6 |
| 144 | Dorico | Italy | A | 83.3 | 87.3 | 80.8 | 89.9 |
| 145 | Genio | Italy | A | 90.0 | 85.7 | 84.4 | 91.8 |
| 146 | Lampo | Italy | A | 77.7 | 73.0 | 73.3 | 79.3 |
| 147 | Libero | Italy | B | 75.0 | 75.7 | 69.7 | 73.3 |
| 148 | Mantol | Italy | B | 77.7 | 66.7 | 73.8 | 75.9 |
| 149 | Sagittario | Italy | A | 82.3 | 85.3 | 79.1 | 81.6 |
| 150 | Kanto 107 | Japan | B | 97.7 | 89.7 | 82.5 | 87.3 |
| 151 | Kitanokaori | Japan | B | 78.3 | 82.0 | 80.5 | 83.2 |
| 152 | Norin 61 | Japan | B | 95.0 | 91.3 | 85.6 | 92.1 |
| 153 | Norin 67 | Japan | A | 91.7 | 90.0 | 87.6 | 94.1 |
| 154 | HK1/6/NVSR3/5/BEZ/TVR/ | Turkey | B | 79.3 | 75.3 | 77.7 | 80.2 |
|  | 5/CFN/BEZ//SU92/CI13645/ |  |  |  |  |  |  |
|  | 3NAI60 |  |  |  |  |  |  |

^a^ A, *FAR-****a***/*AP2-****a*** (JD8 parental type); B, *FAR-****b***/*AP2-****b*** (AK58 parental type);

C, *FAR-****a***/*AP2-****b*** (recombinant type); D, *FAR-****b***/*AP2-****a*** (recombinant type)
