# Supplementary material for: Molecular Mapping of Reduced Plant Height Gene Rht24 in Bread Wheat
Source: Front Plant Sci. 2017 Aug 8;8:1379. doi: 10.3389/fpls.2017.01379 (PMC5550838; doi:10.3389/fpls.2017.01379)
Supplement: Supplementary file 5 [file Table_5.DOCX]

**Supplementary Table** **5** *Rht24* genotypes and plant height of wheat varieties in Set II germplasm

| Code | Variety | Origin | Genotype^a^ | Plant height (cm) | | | |
| --- | --- | --- | --- | --- | --- | --- | --- |
|  |  |  |  | 2012-2013  Beijing | 2012-2013  Shijiazhuang | 2013-2014 Beijing | 2013-2014 Shijiazhuang |
| 1 | Beijing 0045 | Beijing | B | 72.6 | 79.3 | 80.7 | 68.8 |
| 2 | Beijing 841 | Beijing | C | 89.0 | 88.1 | 92.3 | 94.9 |
| 3 | CA0548 | Beijing | B | 72.9 | 75.6 | 75.7 | 77.5 |
| 4 | CA0816 (red) | Beijing | B | 74.1 | 81.3 | 80.0 | 74.9 |
| 5 | CA0816 (white) | Beijing | B | 78.5 | 81.8 | 77.7 | 80.7 |
| 6 | CA0958 | Beijing | B | 78.0 | 77.1 | 92.3 | 83.5 |
| 7 | CA0998 | Beijing | B | 75.0 | 78.9 | 77.0 | 77.1 |
| 8 | CA1055 | Beijing | B | 73.2 | 77.3 | 78.7 | 79.8 |
| 9 | CA1062 | Beijing | B | 69.5 | 74.8 | 76.7 | 69.0 |
| 10 | CA1090 | Beijing | B | 81.7 | 79.3 | 83.3 | 76.1 |
| 11 | CA1119 | Beijing | B | 77.8 | 75.7 | 91.0 | 81.1 |
| 12 | CA1133 | Beijing | B | 73.1 | 77.9 | 75.0 | 75.1 |
| 13 | CA1135 | Beijing | B | 77.8 | 75.0 | 81.7 | 75.6 |
| 14 | CA9719 | Beijing | B | 93.9 | 84.9 | 93.7 | 98.8 |
| 15 | Fengkang 2 | Beijing | A | 81.6 | 84.7 | 84.7 | 83.7 |
| 16 | Jing 411 | Beijing | A | 81.0 | 82.0 | 91.3 | 88.2 |
| 17 | Jing 9428 | Beijing | B | 80.8 | 89.7 | 95.7 | 86.0 |
| 18 | Jingdong 17 | Beijing | B | 73.0 | 79.9 | 80.3 | 75.9 |
| 19 | Jingdong 22 | Beijing | A | 80.9 | 81.7 | 88.0 | 80.1 |
| 20 | Jingdong 8 | Beijing | A | 81.6 | 87.4 | 92.7 | 87.8 |
| 21 | Jingshuang 16 | Beijing | C | 86.0 | 90.8 | 95.7 | 91.3 |
| 22 | Lunxuan 987 | Beijing | B | 78.7 | 88.3 | 77.0 | 74.1 |
| 23 | Nongda 139 | Beijing | A | 102.0 | 97.3 | 101.7 | 97.8 |
| 24 | Nongda 211 | Beijing | B | 74.2 | 79.7 | 88.3 | 74.4 |
| 25 | Nongda 212 | Beijing | B | 79.6 | 80.6 | 80.3 | 74.7 |
| 26 | Zhongmai 175 | Beijing | B | 75.7 | 81.9 | 84.0 | 73.2 |
| 27 | Zhongmai 415 | Beijing | B | 71.3 | 75.9 | 84.3 | 71.8 |
| 28 | Zhongyou 206 | Beijing | B | 75.3 | 78.9 | 90.7 | 87.4 |
| 29 | Zhongyou 335 | Beijing | B | 73.1 | 77.2 | 82.0 | 75.4 |
| 30 | Zhongyou 9507 | Beijing | A | 93.2 | 96.8 | 99.0 | 93.4 |
| 31 | Keheng 6654 | Hebei | D | 67.1 | 72.2 | 79.7 | 69.3 |
| 32 | Doumai | Landrace | A | 78.1 | 76.8 | 84.0 | 81.2 |
| 33 | Yangxiaomai | Landrace | A | 109.8 | 102.0 | 119.3 | 111.8 |
| 34 | Ningdong 10 | Ningxia | B | 90.5 | 94.0 | 100.7 | 88.3 |
| 35 | Ningdong 11 | Ningxia | B | 85.0 | 95.4 | 100.3 | 100.0 |
| 36 | Changwu 134 | Shaanxi | B | 77.6 | 79.3 | 82.0 | 79.3 |
| 37 | Qinnong 142 | Shaanxi | B | 65.2 | 68.7 | 69.3 | 73.3 |
| 38 | Qinnong 151 | Shaanxi | B | 61.2 | 69.4 | 72.3 | 68.7 |
| 39 | Qinnong 731 | Shaanxi | B | 60.8 | 67.1 | 71.0 | 71.1 |
| 40 | Jinmai 45 | Shanxi | B | 73.4 | 76.9 | 71.0 | 67.9 |
| 41 | Jinmai 67 | Shanxi | B | 91.4 | 84.0 | 97.3 | 92.3 |
| 42 | Xinmai 37 | Xinjiang | A | 62.9 | 68.2 | 73.0 | 67.1 |
| 43 | #575(LYFENKO)/JAGGER/ | USA | B | 75.7 | 84.9 | 90.7 | 84.4 |
|  | 4/KARL*2//PI355520/ |  |  |  |  |  |  |
| 44 | Jagger/W94-244-132 | USA | B | 89.9 | 93.0 | 94.0 | 86.2 |
| 45 | Mason/Jagger-1 | USA | A | 88.2 | 88.3 | 90.7 | 89.2 |
| 46 | Mason/Jagger-2 | USA | A | 85.4 | 86.1 | 98.0 | 97.1 |
| 47 | NUWEST/4/D887-74/PEW/3/ | USA | A | 86.8 | 87.0 | 92.3 | 91.3 |
|  | LNCR//CARSTEN/GIGANT/ |  |  |  |  |  |  |
|  | 5/MRS/CI14482//YMH/HYS/ |  |  |  |  |  |  |
|  | 3/RONDEZVOUS |  |  |  |  |  |  |
| 48 | T67/X84W063-9-45//K92/3/ | USA | D | 78.6 | 85.2 | 90.0 | 88.5 |
|  | SNF/4/X86509-1-1/X84W063- |  |  |  |  |  |  |
|  | 9-39-2//K93 |  |  |  |  |  |  |
| 49 | TX03A0148 | USA | D | 82.8 | 80.9 | 84.7 | 75.7 |
| 50 | WGRC10/3/KS93U69 sib/ | USA | A | 81.8 | 84.8 | 88.7 | 78.5 |
|  | TA2455//KS93U69/4/JAGGER |  |  |  |  |  |  |
| 51 | C39 | UK | B | 73.7 | 83.1 | 81.3 | 79.8 |
| 52 | Aztec | France | A | 81.6 | 82.2 | 88.0 | 83.4 |
| 53 | Azulon | France | B | 79.0 | 87.2 | 88.7 | 88.6 |
| 54 | BRUTA | France | A | 68.8 | 77.6 | 112.5 | 104.3 |
| 55 | Carimulti | France | B | 88.7 | 95.4 | 95.7 | 98.9 |
| 56 | Darius | France | B | 83.2 | 87.0 | 90.3 | 93.6 |
| 57 | Festin | France | D | 80.8 | 80.7 | 89.0 | 91.1 |
| 58 | Fr03711 | France | B | 89.6 | 91.8 | 94.7 | 106.5 |
| 59 | Fr03717 | France | A | 67.7 | 70.4 | 72.7 | 72.1 |
| 60 | Fr03724 | France | B | 68.7 | 74.6 | 74.3 | 73.7 |
| 61 | Fr03725 | France | B | 65.9 | 74.1 | 71.3 | 76.0 |
| 62 | Fr03732 | France | A | 82.0 | 82.3 | 83.7 | 81.7 |
| 63 | Fr03733 | France | B | 76.3 | 80.8 | 83.7 | 84.1 |
| 64 | Insignia | France | A | 72.3 | 79.7 | 72.3 | 75.3 |
| 65 | lasen-1 | France | B | 70.1 | 72.3 | 77.7 | 89.7 |
| 66 | lasen-2 | France | B | 71.9 | 80.8 | 85.0 | 67.7 |
| 67 | Manital | France | B | 49.3 | 55.6 | 66.0 | 59.8 |
| 68 | Mesofold | France | B | 71.4 | 73.8 | 85.3 | 74.0 |
| 69 | NSA09-3645 | France | B | 66.1 | 71.6 | 71.0 | 73.4 |
| 70 | Soissons | France | B | 73.1 | 77.7 | 77.3 | 83.7 |
| 71 | Thesee | France | B | 73.7 | 85.6 | 86.7 | 81.3 |
| 72 | YANA | France | A | 83.4 | 84.8 | 90.3 | 86.1 |
| 73 | Magnus | Germany | B | 90.1 | 97.3 | 90.0 | 88.2 |
| 74 | MV LAURA | Hungary | A | 84.4 | 88.7 | 82.0 | 89.8 |
| 75 | MV05-08 | Hungary | B | 72.8 | 73.7 | 90.7 | 84.4 |
| 76 | RE714 | Norway | B | 78.3 | 81.0 | 80.7 | 82.0 |
| 77 | 98039G5-103 | Romania | A | 75.0 | 79.6 | 79.0 | 76.0 |
| 78 | F498U1-1021 / BOEMA | Romania | B | 72.2 | 79.0 | 83.7 | 81.0 |
| 79 | F92080G1-1/F93042G2-1 | Romania | A | 80.9 | 85.7 | 90.0 | 91.4 |
| 80 | F98047G14-2INC | Romania | B | 82.8 | 84.0 | 89.3 | 82.1 |
| 81 | Lovrin10 | Romania | A | 91.6 | 108.4 | 104.3 | 103.4 |
| 82 | Lovrin13 | Romania | B | 82.7 | 88.0 | 88.7 | 87.2 |
| 83 | BATJKO | Russia | B | 73.7 | 81.8 | 88.7 | 83.6 |
| 84 | DONSKI-93 | Russia | B | 79.3 | 85.2 | 82.7 | 83.0 |
| 85 | KNIISH 46 | Russia | C | 70.9 | 78.9 | 75.7 | 72.7 |
| 86 | PALPICH | Russia | B | 72.8 | 77.1 | 78.3 | 75.2 |
| 87 | SELYANKA | Russia | B | 81.8 | 85.7 | 88.7 | 82.8 |
| 88 | STARSHINA | Russia | A | 73.9 | 84.8 | 84.7 | 81.3 |

^a^ A, *FAR-****a***/*AP2-****a*** (JD8 parental type); B, *FAR-****b***/*AP2-****b*** (AK58 parental type);

C, *FAR-****a***/*AP2-****b*** (recombinant type); D, *FAR-****b***/*AP2-****a*** (recombinant type)
