# Supplementary material for: Molecular Mapping of Reduced Plant Height Gene Rht24 in Bread Wheat
Source: Front Plant Sci. 2017 Aug 8;8:1379. doi: 10.3389/fpls.2017.01379 (PMC5550838; doi:10.3389/fpls.2017.01379)
Supplement: Supplementary file 6 [file Table_6.DOCX]

**Table S6** Analysis of variance and broad-sense heritability for plant height in RILs based on two environments.

| Source of variation | Df | Mean of squares | F values | *h_B_^2^* |
| --- | --- | --- | --- | --- |
| Line | 255 | 398.67 | 8.59*** | 0.87 |
| Environment | 1 | 125,033.68 | 2692.76*** |  |
| Replicate | 2 | 62.84 | 1.35 |  |
| Line×environment | 255 | 52.54 | 1.13 |  |
| Error | 1022 | 46.43 |  |  |
