# Supplementary material for: Molecular Mapping of Reduced Plant Height Gene Rht24 in Bread Wheat
Source: Front Plant Sci. 2017 Aug 8;8:1379. doi: 10.3389/fpls.2017.01379 (PMC5550838; doi:10.3389/fpls.2017.01379)
Supplement: Supplementary file 7 [file Table_7.DOCX]

**Supplementary Table 7** SSR markers used to map *Rht24*

| SSR marker | Forward primer (5'-3') | Reverse primer (5'-3') | Target fragment size (bp) |
| --- | --- | --- | --- |
| *Xbarc3* | TTCCCTGTGTCTTTCTAATTTTTTTT | GCGAACTCCCGAACATTTTTAT | 193 |
| *Xbarc48* | GCGAGCTGCAGAGGTCCATC | GCGTTAGTCTTCTTGGTCAATCAC | 164 |
| *Xbarc103* | GCTCGGTACCCAGTTCATCGAATGTA | GCGAATGTTGAGTGGCTCTCATTTGA | 224 |
| *Xbarc146* | AAGGCGATGCTGCAGCTAAT | GGCAATATGGAAACTGGAGAGAAAT | 163 |
| *Xbarc195* | CCCACATGTCATTGGCTGTTTAA | GCCCGGCCCAGAACGATTTAAATG | 223 |
| *Xgwm132* | TACCAAATCGAAACACATCAGG | CATATCAAGGTCTCCTTCCCC | 115 |
| *Xgwm494* | ATTGAACAGGAAGACATCAGGG | TTCCTGGAGCTGTCTGGC | 204 |
| *Xgwm570* | TCGCCTTTTACAGTCGGC | ATGGGTAGCTGAGAGCCAAA | 149 |
| *Xwmc145* | GGCGGTGGGTTCAAGTCGTCTG | GGACGAGTCGCTGTCCTCCTGG | 283 |
| *Xwmc150* | CATTGATTGAACAGTTGAAGAA | CTCAAAGCAACAGAAAAGTAAA | 258 |
| *Xwmc179* | CATGGTGGCCATGAGTGGAGGT | CATGATCTTGCGTGTGCGTAGG | 241 |
| *Xwmc201* | CATGCTCTTTCACTTGGGTTCG | GCGCTTGCAGGAATTCAACACT | 250 |
| *Xwmc243* | CGTCATTTCCTCAAACACACCT | ACCGGCAGATGTTGACAATAGT | 167 |
| *Xwmc256* | CCAAATCTTCGAACAAGAACCC | ACCGATCGATGGTGTATACTGA | 174 |
| *Xwmc398* | GGAGATTGACCGAGTGGAT | CGTGAGAGCGGTTCTTTG | 132 |
| *Xwmc553* | CGGAGCATGCAGCTAGTAA | CGCCTGCAGAATTCAACAC | 358 |
| *Xwmc672* | GGAGGAGCAAGCTAGGCAA | TTTATAGAGGGAGGGGAGGCAG | 98 |
| *Xwmc684* | CGAATCCAACGAGGCCATAGA | GCAATCAGGAGGCATCCACC | 187 |
| *Xwmc748* | CCAGCCCAGATGCTTCAATG | ACGTGGGTGCAATTCTCAGG | 172 |
| *Xwmc753* | AAGGTGAAGATGATGCTCGC | TGACTGATCATGGATTGCCC | 322 |
| *Xwmc786* | GGGTCACCAACCCGCTC | CGTGGGTGCAATTCTCAGG | 188 |
| *Xwmc807* | ATCCAACAAGGCCTCACCAT | GCAGGTTTGATCTGGATTTCATC | 224 |
