# Supplementary material for: Molecular Mapping of Reduced Plant Height Gene Rht24 in Bread Wheat
Source: Front Plant Sci. 2017 Aug 8;8:1379. doi: 10.3389/fpls.2017.01379 (PMC5550838; doi:10.3389/fpls.2017.01379)
Supplement: Supplementary file 8 [file Table_8.DOCX]

**Supplementary Table 8** Genes from the annotated super-scaffold for developing molecular markers

| Code | Temporary name | Accession number | Annotation | Polymorphism |
| --- | --- | --- | --- | --- |
| 1 | *TaHY5* | Traes_6AS_AFF47E71C | Transcription factor HY5 | No |
| 2 | *TaGRAS* | Traes_6AL_4084532FC1 | GRAS domain family | No |
| 3 | *TaGA3* | Traes_6AL_751C1A39D | Gibberellin 3-beta-dioxygenase | Yes |
| 4 | *TaAuxin* | Traes_6AS_10D92215D.2 | Auxin response factor 6 | No |
| 5 | *TaFAR* | Traes_6AL_1DC1EF368.2 | Protein FAR-red elongated hypocotyl 3 | Yes |
| 6 | *TaCytokinin8* | Traes_6AS_4B78E3665.1 | Cytokinin oxidase/dehydrogenase 8 | No |
| 7 | *TaAP2* | Traes_6AL_EE7541F47.1 | AP2-like ethylene-responsive transcription factor BBM2 | Yes |
| 8 | *TaSBP* | Traes_6AS_32A35158A.1 | Squamosa-Promoter binding protein | No |
| 9 | *TaTPA* | Traes_6AS_E8AEEB7F7.1 | Cell division protein | No |
| 10 | *TaEF1G* | Traes_6AS_91194988A.2 | Elongation factor 1-gamma 2 | No |
| 11 | *TaMadx* | Traes_6AS_9AA76345D.1 | MADS-box transcription factor 16 | No |
| 12 | *TaCyclin* | Traes_6AL_54C680E88.1 | Cyclin-dependent kinase G-1 | No |
| 13 | *TaGUB* | Traes_6AL_FB41DAA2A.1 | Wall-associated receptor kinase 5 | No |
| 14 | *TaPTZ* | Traes_6AS_658FD4BB5.1 | cyclin-dependent protein kinase | No |
| 15 | *TaCytokinin3* | Traes_6AS_A88CB01FD.1 | Cytokinin-O-glucosyltransferase 3 | No |
| 16 | *TaSTK* | Traes_6AS_E690A27CA.1 | Serine/threonine-protein kinase fray2 | No |
| 17 | *TaGTE4* | Traes_6AS_5C6CBFDEF.2 | Transcription factor GTE4 | No |
| 18 | *TaElongation* | Traes_6AS_91194988A.2 | Elongation factor 1-gamma 2 | No |
| 19 | *TaWIN1* | Traes_6AS_8E7166D79.1 | Ethylene-responsive transcription factor WIN1 | No |
| 20 | *TaFtsh* | Traes_6AS_7ACD8E491.1 | Cell division protein | No |
