# Supplementary material for: Molecular Mapping of Reduced Plant Height Gene Rht24 in Bread Wheat
Source: Front Plant Sci. 2017 Aug 8;8:1379. doi: 10.3389/fpls.2017.01379 (PMC5550838; doi:10.3389/fpls.2017.01379)
Supplement: Supplementary file 9 [file Table_9.DOCX]

**Supplementary Table 9** SNPs from 660K SNP chip for developing molecular markers

| Code | Temporary name | Probe Set ID | Accession number | Annotation |
| --- | --- | --- | --- | --- |
| 1 | *TaSNP1* | AX-95659865 | Traes_6AS_2941E06A0 | Eps15 homology domain; GTPase era |
| 2 | *TaSNP2* | AX-109854459 | Traes_6AL_BC88BF232 | Cytosolic Fe-S cluster assembly factor NUBP1-like protein |
| 3 | *TaSNP3* | AX-109882821 | Traes_6AL_58B6B7319 | Bidirectional sugar transporter SWEET4 |
| 4 | *TaSNP4* | AX-111183446 | Traes_6AS_A137671B1 | Rhodanese homology domain |
| 5 | *TaSNP5* | AX-86176819 | Traes_6AS_42E1FB04C | Calcineurin-like phosphoesterase superfamily domain |
| 6 | *TaSNP6* | AX-109854459 | Traes_6AL_BC88BF232 | Cell division ATPase MinD |
| 7 | *TaSNP7* | AX-111080243 | Traes_6AL_FB41DAA2A | Wall-associated receptor kinase 5 |
| 8 | *TaSNP8* | AX-111164969 | Traes_6AL_808936F9E | Adenine phosphoribosyltransferase |
| 9 | *TaSNP9* | AX-109882821 | Traes_6AL_58B6B7319 | Phosphoribosyl transferase (PRT)-type I domain |
| 10 | *TaSNP10* | AX-109019106 | Traes_6AL_077524EE1 | ABA 8'-hydroxylase |
