# Supplementary material for: Molecular Mapping of Reduced Plant Height Gene Rht24 in Bread Wheat
Source: Front Plant Sci. 2017 Aug 8;8:1379. doi: 10.3389/fpls.2017.01379 (PMC5550838; doi:10.3389/fpls.2017.01379)
Supplement: Supplementary file 10 [file Image_1.PDF]

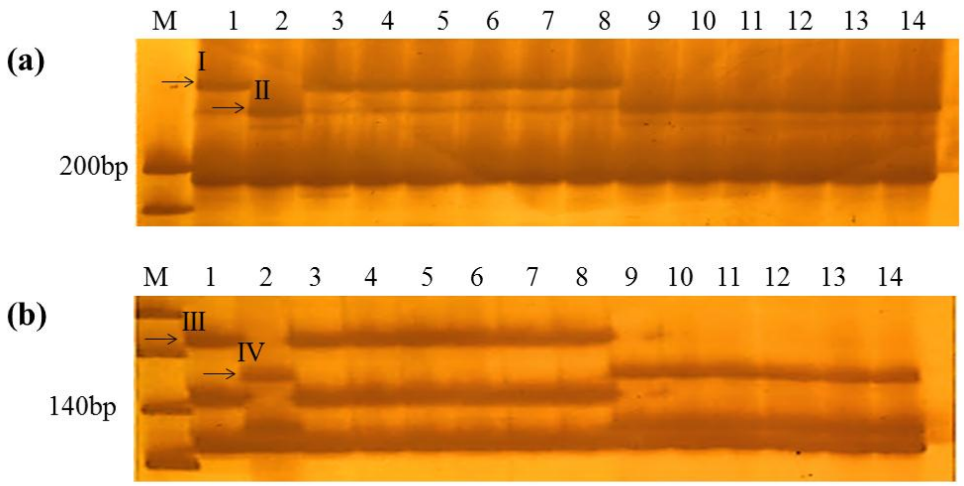

Supplementary Image 1 PCR amplification patterns of *Xbarc103* (a) and *Xwmc256* (b). Fragments polymorphic between short and high lines are indicated by *arrows*. M, Marker (20 bp DNA ladder, Takara Bio Co.). *Lanes 1* and *2* are the parents, AK58 and JD8, respectively; *lanes 3–8*, short RILs; *lanes 9–14*, tall RILs; I, II, III and IV are the target fragments.
