# Supplementary material for: Molecular Mapping of Reduced Plant Height Gene Rht24 in Bread Wheat
Source: Front Plant Sci. 2017 Aug 8;8:1379. doi: 10.3389/fpls.2017.01379 (PMC5550838; doi:10.3389/fpls.2017.01379)
Supplement: Supplementary file 11 [file Image_2.pdf]

(a)

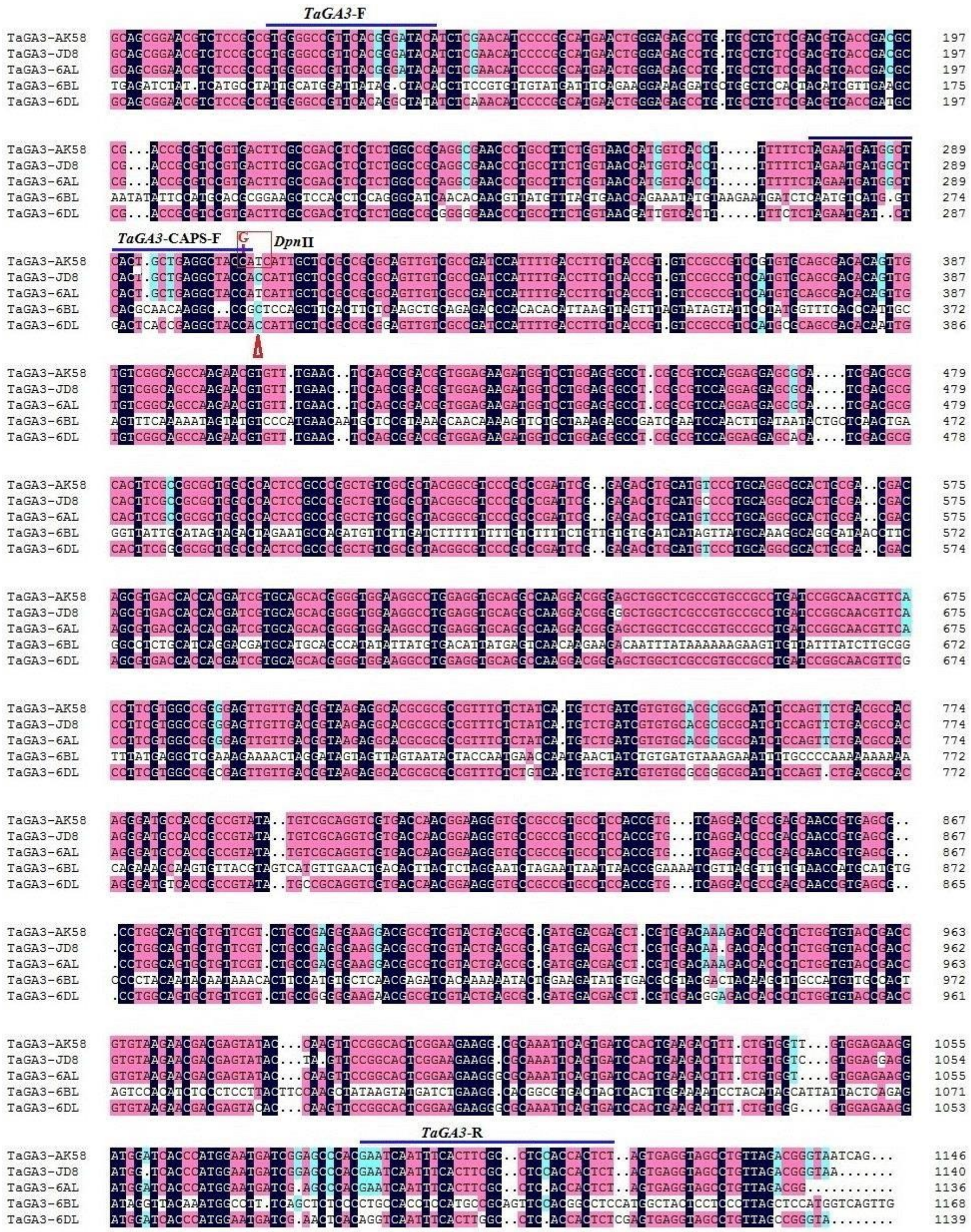
TaGA3-F
TaGA3-CAPS-F
G
DpnII
TaGA3-R
TaGA3-AK58
TaGA3-JD8
TaGA3-6AL
TaGA3-6BL
TaGA3-6DL

(b)

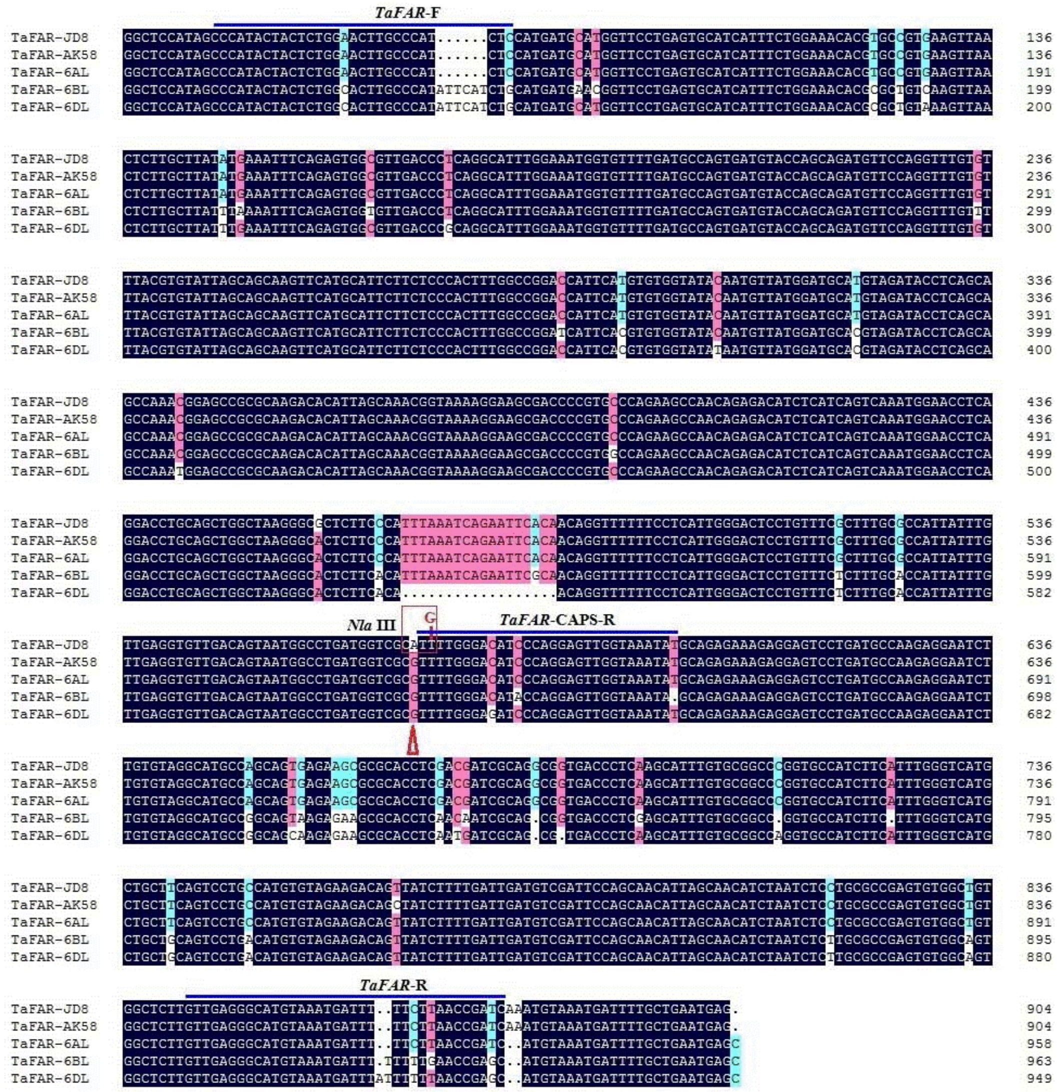

TaFAR-F
Nla III
G
TaFAR-CAPS-R
TaFAR-R
TaFAR-JD8
TaFAR-AK58
TaFAR-6AL
TaFAR-6BL
TaFAR-6DL

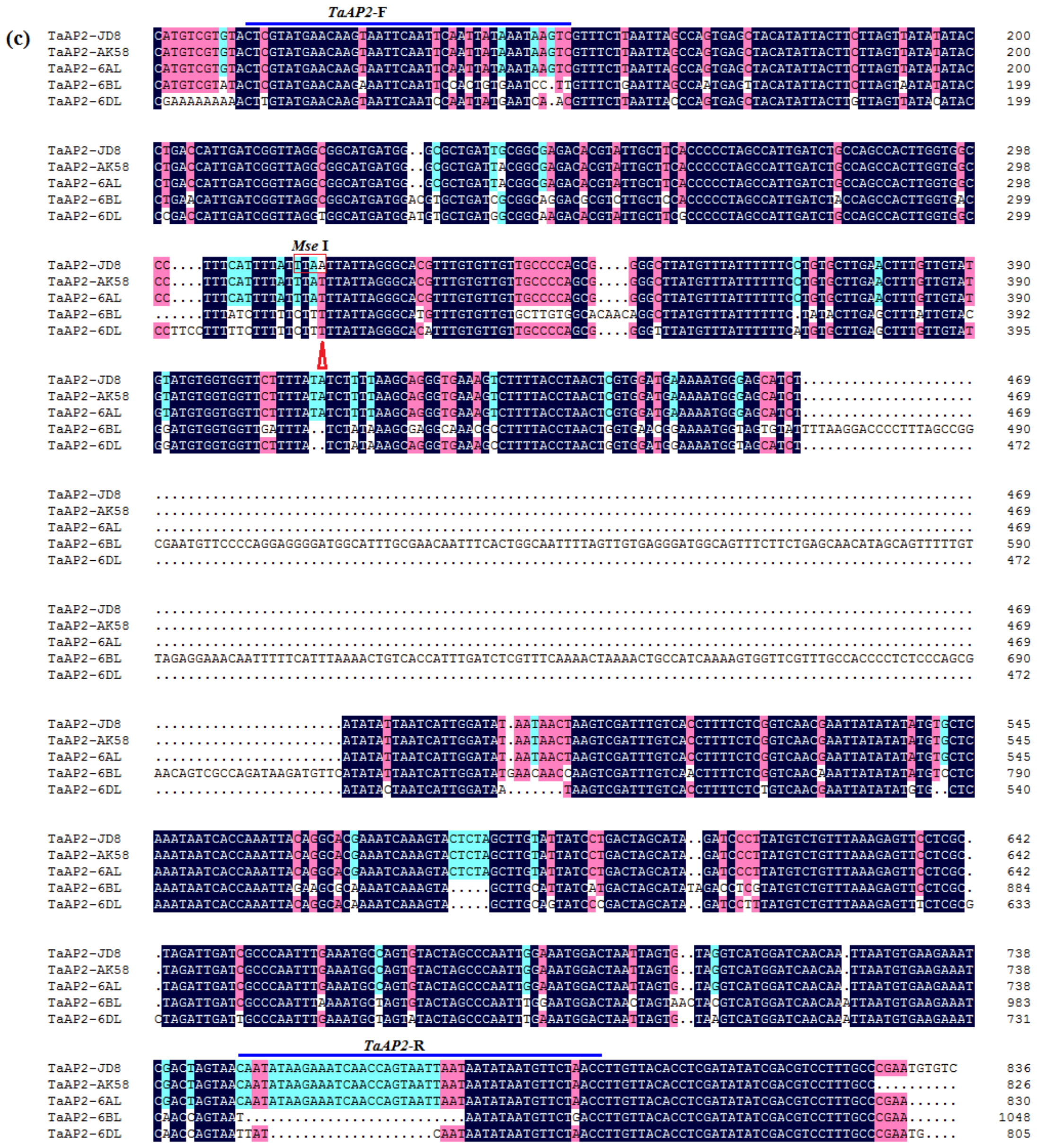

Fig. S2 Nucleotide sequence alignments for *TaGA3* (a), *TaFAR* (b) and *TaAP2* (c) cloned from AK58 and JD8 using the appropriate primer pairs.

(a) The primer pair *TaGA3*-CAPS-F/*TaGA3*-R was used to developed a CAPS marker. The arrow shows the target site of the SNP and the box shows the position of the restriction site for the CAPS marker. (b) The primer pair *TaFAR*-F/*TaFAR*-CAPS-R was used to developed a CAPS marker. The arrow shows the target site of the SNP and the box shows the position of restriction site for the CAPS marker. (c) The primer pair *TaAP2*-F/*TaAP2*-R was also used to develop a CAPS marker. Arrow shows the target site of the SNP and the box shows the position of restriction site for the CAPS

marker
