# Supplementary material for: Molecular Mapping of Reduced Plant Height Gene Rht24 in Bread Wheat
Source: Front Plant Sci. 2017 Aug 8;8:1379. doi: 10.3389/fpls.2017.01379 (PMC5550838; doi:10.3389/fpls.2017.01379)
Supplement: Supplementary file 12 [file Image_3.PDF]

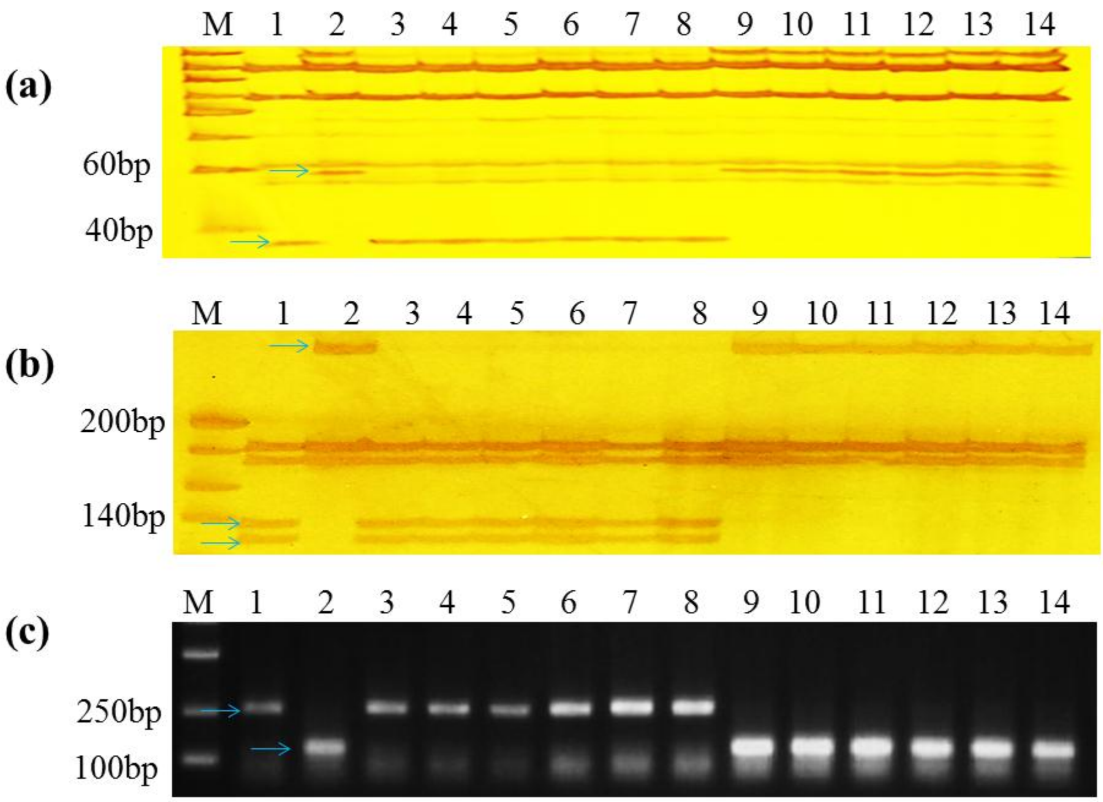

Supplementary Image 3 PCR patterns of polymorphic markers, *TaSNP1* (a), *TaSNP2* (b) and *TaSNP3* (c). M, Marker (20 bp DNA ladder, Takara Bio Company). Target fragment that are polymorphic between short and tall lines are shown with *arrows*. *Lanes 1* and *2*, parents, AK58 and JD8, respectively; *lanes* 3–8 are short RILs; *lanes* 9–14 are tall RILs.
