# Supplementary material for: Molecular Mapping of Reduced Plant Height Gene Rht24 in Bread Wheat
Source: Front Plant Sci. 2017 Aug 8;8:1379. doi: 10.3389/fpls.2017.01379 (PMC5550838; doi:10.3389/fpls.2017.01379)
Supplement: Supplementary file 13 [file Image_4.PDF]

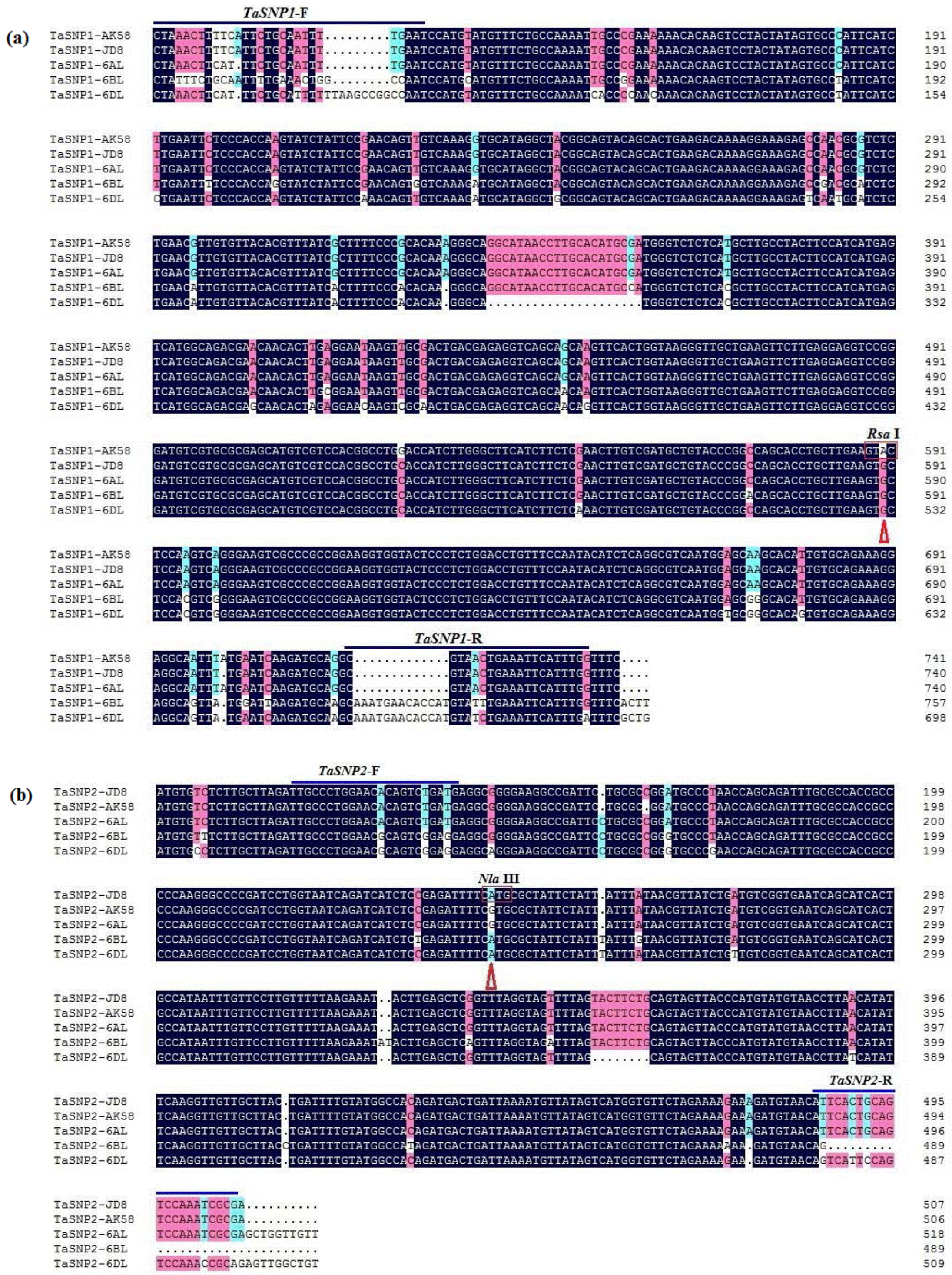
(a)
TaSNP1-F
TaSNP1-AK58
TaSNP1-JD8
TaSNP1-6AL
TaSNP1-6BL
TaSNP1-6DL
Rsa I
TaSNP1-R
(b)
TaSNP2-F
TaSNP2-JD8
TaSNP2-AK58
TaSNP2-6AL
TaSNP2-6BL
TaSNP2-6DL
Nla III
TaSNP2-R

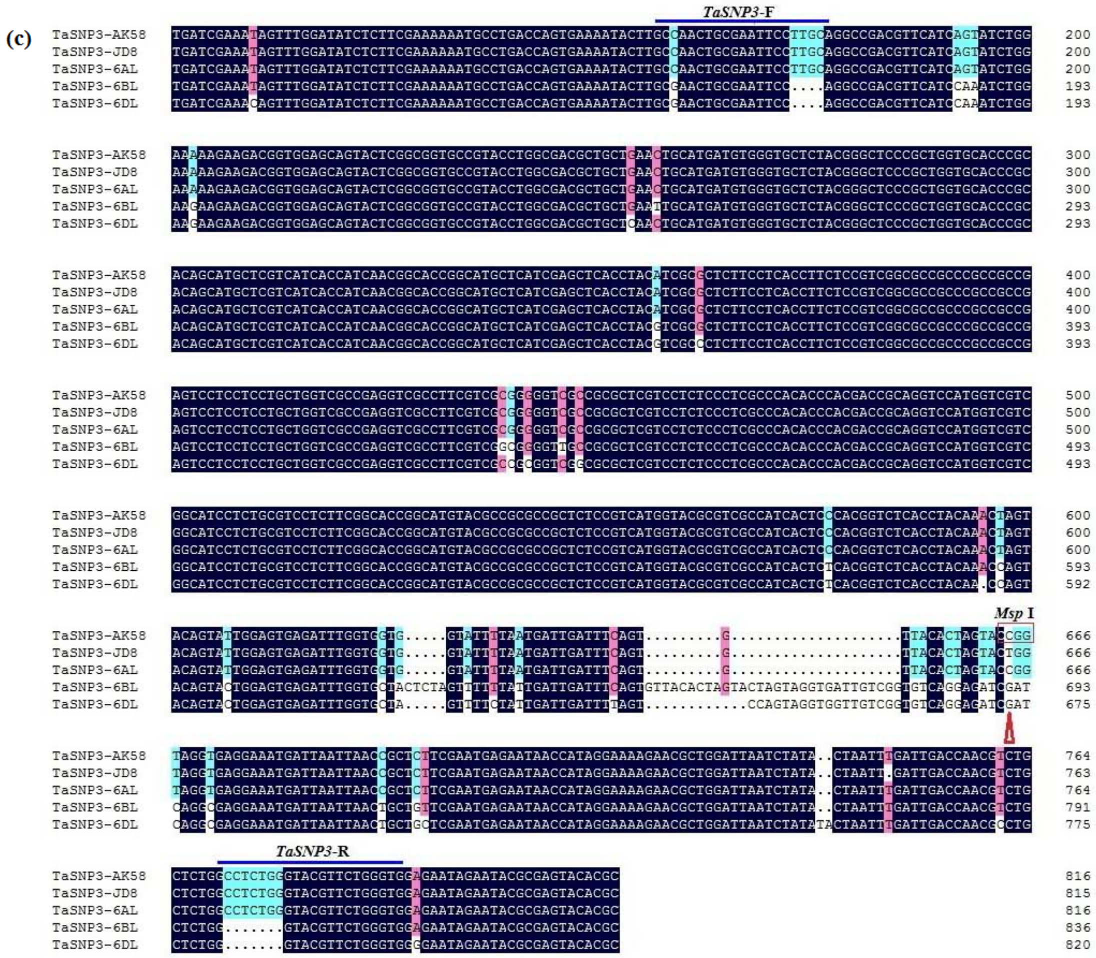

**Supplementary Image 4** Nucleotide sequence alignments for *TaSNP1* (a), *TaSNP2* (b) and *TaSNP3* (c) cloned from AK58 and JD8 using the appropriate primer pairs. (a) The primer pair *TaSNP1*-F/*TaSNP1*-R was used to develop a CAPS marker. The arrow shows the target site of SNP and the box shows the position of restriction site for the CAPS marker. (b) The primer pair *TaSNP2*-F/*TaSNP2*-R was used to develop CAPS marker. The arrow shows the target site of SNP and the box shows the position of restriction site for the CAPS marker. (c) The primer pair *TaSNP3*-F/*TaSNP3*-R was also used to develop a CAPS marker. The arrow shows the target site of SNP and the box shows the position of restriction site for the CAPS marker.
