# Supplementary material for: Molecular Mapping of Reduced Plant Height Gene Rht24 in Bread Wheat
Source: Front Plant Sci. 2017 Aug 8;8:1379. doi: 10.3389/fpls.2017.01379 (PMC5550838; doi:10.3389/fpls.2017.01379)
Supplement: Supplementary file 14 [file Image_5.PDF]

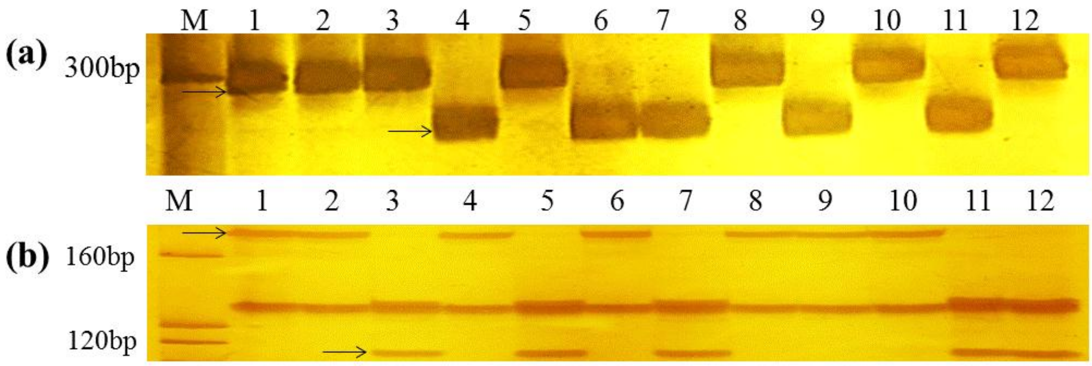

Supplementary Image 5 Amplification patterns of markers *TaFAR* (a) and *TaAP2* (b) in the representative Chinese elite wheat varieties. M, Marker (20 bp DNA ladder, Takara Bio Co.). *Lanes* 1–12, Jimai 20, Zhongmai 895, Shijiazhuang 15, Jingshuang 16, Gaoyou 503, Lumai 5, Jingdong 5, Bainong 64, Xiaoyan 6, Xiaoyan 54, Yannong 18 and Zhongyu 9, respectively.
